# Mutant *TP53* allelic states in human hematopoietic stem and progenitor cells

**DOI:** 10.1038/s41375-026-03106-x

**Published:** 2026-08-18

**Authors:** Karin Lind, Sayantanee Dutta, Marco Raffaele Cosenza, Thomas Eder, Johannes Foßelteder, Gabriel Pabst, Wolfgang Schöll, Andreas Prokesch, Holger W. Auner, Albert Wölfler, Armin Zebisch, Andreas Reinisch, Florian Grebien, Jan O. Korbel, Heinz Sill

**Affiliations:** 1 Division of Hematology, Medical University of Graz, Graz, Austria; 2 Division of Oncology, Medical University of Graz, Graz, Austria; 3 Genome Biology Unit, European Molecular Biology Laboratory, Heidelberg, Germany; 4 Department of Biological Sciences and Pathobiology, University of Veterinary Medicine Vienna, Vienna, Austria; 5 Department of Obstetrics & Gynecology, Medical University of Graz, Graz, Austria; 6 Gottfried Schatz Research Center for Cell Signaling, Metabolism and Aging, Division of Cell Biology, Histology and Embryology, Medical University of Graz, Graz, Austria; 7 Division of Haematology and Central Haematology Laboratory, Lausanne University Hospital, Lausanne, Switzerland; 8 Otto Loewi Research Center for Vascular Biology, Immunology and Inflammation, Division of Pharmacology, Medical University of Graz, Graz, Austria; 9 Department of Blood Group Serology and Transfusion Medicine, Medical University of Graz, Graz, Austria; 10 St. Anna Children’s Cancer Research Institute (CCRI), Vienna, Austria; 11 CeMM Research Center for Molecular Medicine of the Austrian Academy of Sciences, Vienna, Austria; 12 Bridging Research Division on Mechanisms of Genomic Variation and Data Science, German Cancer Research Center (DKFZ), Heidelberg, Germany

**Keywords:** Cancer stem cells, Cancer genomics

## To the Editor:

In human hematopoietic stem and progenitor cells (HSPCs), *TP53* mutations characterize clonal hematopoiesis of indeterminate potential (CHIP) and are recognized as a high-risk factor for the development of non-neoplastic and neoplastic disorders, particularly myelodysplastic syndromes (MDS) and acute myeloid leukemia [1, 2]. During leukemic transformation, the remaining *TP53* wild-type (WT) allele is frequently affected, most commonly through loss-of-heterozygosity (LOH), copy-neutral LOH, or a second concurrent mutation. The *TP53* allelic state—reflecting whether one or both alleles are affected—remains a subject of debate regarding its prognostic relevance in patients with MDS [3]. Recently, the biological consequences of monoallelic versus biallelic aberrations were investigated using a Trp53 mouse model [4]. Here, we address this question in human HSPCs by introducing defined *TP53* aberrations via CRISPR/Cas9-mediated genome editing.

**Fig. 1:**
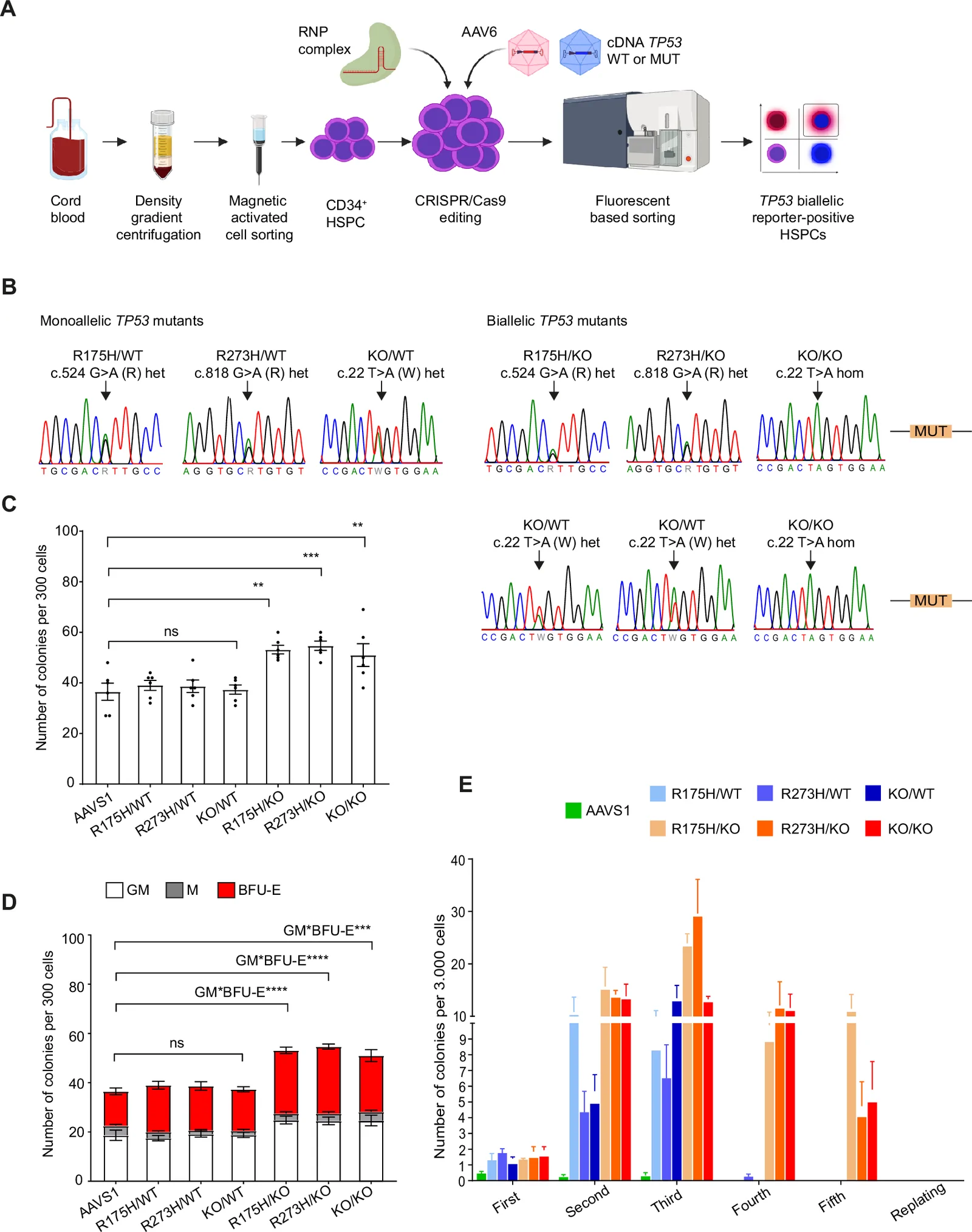
The *TP53* allelic state differentially modulates human HSPC growth and self-renewal. **A** CRISPR-mediated knock-in of wild-type (WT) or mutant (MUT) *TP53* cDNA into cord blood-derived CD34⁺ HSPCs. **B** Representative genomic DNA chromatograms displaying monoallelic (R175H/WT, R273H/WT, KO/WT) and biallelic (R175H/KO, R273H/KO, KO/KO) *TP53* genotypes. **C** CFUs from different genotypes 14 days after seeding into methylcellulose. **D** Morphological classification of colonies as BFU-E (red), monocyte (M, gray) or granulocyte/macrophage (GM, white) from primary plating after 14 days. **E** CFUs from different genotypes upon replating. **C**–**E** Data are presented as mean ± s.e.m. of two pooled samples plated in triplicates. Statistical comparisons to *AAVS1* control by one-way (**C**) or two-way (**D**) ANOVA. ns not significant; *P < 0.05; **P < 0.01; ***P < 0.001; ****P < 0.0001. HSPCs hematopoietic stem and progenitor cells, CFUs colony-forming units, BFU-E burst-forming unit erythroid.

Material and methods are described in detail in the *Supplementary information*. Enriched, umbilical cord blood (UCB) derived CD34^+^ HSPCs were targeted at exon 2 of the *TP53* gene using a CRISPR/Cas9 and recombinant adeno-associated virus serotype 6 mediated knock-in strategy (Fig. 1A and Supplementary Figs. S1A, B and S2) [5]. By targeting exon 2, we were able to generate WT and aberrant *TP53* alleles within the same genomic context while maintaining the endogenous regulatory control. Integration of the donor template at exon 2 disrupts the endogenous *TP53* gene, ensuring that only the donor-encoded cDNA sequences are expressed from the endogenous *TP53* promoter. Mutant *TP53* cDNA—carrying either the variant of interest or a stop codon—or WT cDNA, each linked to a fluorescent reporter (FR) expression cassette, was introduced via homology-directed repair to generate the following monoallelic and biallelic genotypes: *TP53*^R175H/WT^, *TP53*^R273H/WT^, *TP53*^KO/WT^ and *TP53*^R175H/KO^, *TP53*^R273H/KO^, *TP53*^KO/KO^. The p.R175H and p.R273H aberrations are *TP53* hot-spot mutations with reported gain-of-function and dominant-negative/loss-of-function properties, respectively (Supplementary Table S1) [6–8]. FR inserted at the A*AVS1* safe-harbor locus served as *TP53*^WT/WT^ control. Stable, biallelic integration of *TP53* cDNAs was achieved in 0.5–10.0% of HSPCs (mean, 2.23 ± 1.76 SD) and enriched by flow cytometry (Supplementary Fig. 1C). Site-specific integration was confirmed by PCR and each engineered *TP53* genotype was validated by Sanger sequencing of both genomic DNA and complementary DNA (Fig. 1B and Supplementary Fig. 3A, B), p53 protein expression was confirmed by immunocytochemistry (Supplementary Fig. 3C). Functionally, the introduced *TP53* aberrations resulted in reduced or absent expression of the downstream targets p21 and MDM2 (Supplementary Fig. S4).

We initially investigated whether distinct *TP53* mutant allelic states affect colony growth and self-renewal using methylcellulose-based colony-forming unit (CFU) assays. Reporter-positive, genome-edited HSPCs with biallelic *TP53* aberrations derived from pooled UCB samples generated significantly more colonies than cells with monoallelic aberrations and *AAVS1* controls (Fig. 1C, D). Both CFU-GM and BFU-E colony numbers increased, with erythroid colonies exhibiting a particularly pronounced rise. PCR and Sanger sequencing of colonies showed complete retention of the initially introduced mutations in HSPCs (Supplementary Fig. S5). Serial replating CFU assays demonstrated that HSPCs with WT *TP53* or monoallelic aberrations lost their colony-forming capacity after three passages, whereas cells with biallelic aberrations displayed elevated clonogenic potential (Fig. 1E). Cell cycle analysis revealed a non-significant increase in the proportion of cells with biallelic *TP53* aberrations in the S-phase of the cell cycle (Supplementary Fig. S6).

**Fig. 2:**
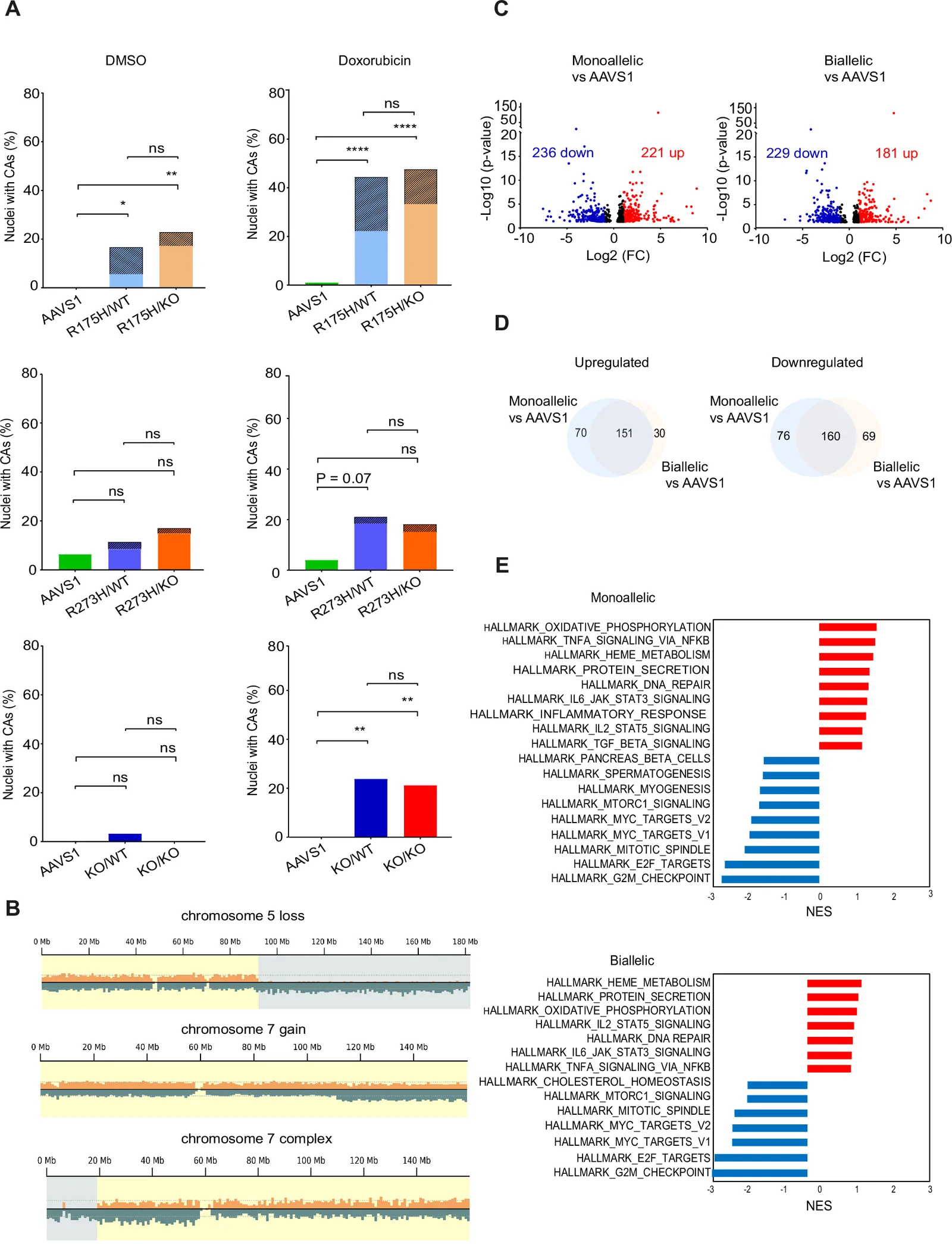
*TP53* mutations in human HSPCs drive chromosomal aberrations (CAs) and activate inflammatory transcriptional programs. **A** Frequency of CAs in *TP53* mutant HSPCs treated with DMSO or doxorubicin; shaded areas of the bars indicate multiple CAs per single nucleus. Statistical comparisons to *AAVS1* controls were performed using two-sided Fisher’s exact test. Data were generated from one biological donor per genotype. **B** Representative CA plots for chromosome 5 loss, chromosome 7 gain and complex aberrations on chromosome 7 with sequencing reads aligned to the positive (Watson, orange) or negative (Crick, green) strands. **C** Volcano plots depicting differentially expressed genes (DEGs) in monoallelic and biallelic *TP53* mutants versus *AAVS1* controls. **D** DeepVenn diagrams showing DEG overlaps. (E) Gene set enrichment analysis (MSigDB hallmark gene sets) highlighting relevant enriched pathways (FDR < 0.25); positive normalized enrichment scores (NES) in red, negative NES in blue. **C**–**E** Data were generated from three independent biological donor-derived HSPC pools.

*TP53* aberrations are closely linked to structural chromosomal abnormalities (CAs). Using single-cell template strand sequencing of genome-edited HSPCs derived from single UCB donors, we analyzed CAs induced by *TP53* aberrations [9]. Cells were treated with either doxorubicin, a potent cellular stressor, or DMSO as control (Supplementary Figs. S7, S8). Upon doxorubicin treatment, cells with *TP53* monoallelic aberrations already exhibited a significantly higher frequency of CAs compared to *AAVS1*-targeted controls. In addition, a spontaneous and significant increase in CAs was observed in HSPCs carrying the *TP53*^R175H^ mutation (Fig. 2A, Supplementary Fig. S9). In HSPCs harboring the *TP53* R175H and R273H mutations, chromosomes 3, 5, 7 and 17 were among the most frequently affected. Aberrations of chromosomes 5 and 7 are also common in patients with *TP53* mutated myeloid neoplasms. Consistent with the clinical situation, we observed terminal losses (64% of all CAs affecting chromosomes 5 and 7), terminal gains (14%), arm gains (7%), whole chromosome gains (7%) and complex aberrations (8%) in our experimental setting (Fig. 2B, Supplementary Fig. S10).

To delineate the transcriptional consequences of monoallelic and biallelic *TP53* aberrations, we performed RNA sequencing on reporter-positive, genome-edited cells from pooled UCB samples. Compared with *AAVS1* controls, the majority of differentially expressed genes (DEGs) were shared between both *TP53* aberrant groups (Fig. 2C, D). Among uniquely expressed genes, monoallelic *TP53* aberrant HSPCs—in particular those with the R175H and R273H genotypes—showed upregulation of PD-L1 (CD274) which was confirmed by flow cytometry (Supplementary Fig. S11). Biallelic *TP53* aberrations were associated with overexpression of HOXA10 (Supplementary Tables S2 and S3). Gene Set Enrichment Analysis of shared DEGs highlighted upregulation of oxidative phosphorylation, heme metabolism and inflammatory signaling including TNFα/NF-κB, IL6-JAK-STAT3 and IL2-STAT5 (Supplementary Fig. S12), whereas downregulated genes were enriched for mitotic spindle assembly and G_2_M checkpoint regulation (Fig. 2E). Furthermore, we observed negative enrichment of a set of 135 myeloid-specific genes in HSPCs carrying either monoallelic or biallelic *TP53* aberrations, while 100 erythroid-associated genes were positively enriched in both groups compared to T*P53* WT HSPCs. The ratio of CEBPA to erythroid-specific transcripts such as GATA1 and PLIN2 was reduced in both monoallelic and biallelic mutant HSPCs, consistent with a shift in lineage commitment as we have observed in the CFU assays (Supplementary Fig. S13).

In this study, we established a model of mutant *TP53* allelic states in human HSPC using CRISPR/Cas9 genome engineering. By employing UCB-derived cells, we were able to investigate the functional consequences of particular *TP53* aberrations without confounding effects of an aging cellular microenvironment. We found that genomic aberrations can be triggered by a single *TP53* aberration, consistent with dysregulation of mitotic spindle and G_2_M checkpoints, as revealed by transcriptome analysis. The spectrum of CAs mirrors that observed in *TP53-*mutated myeloid malignancies indicating that these lesions constitute early events in leukemogenesis. Notably, the R175H mutation led to a spontaneous rise in CAs, supporting its previously described gain-of-function activity in acute leukemia models [6]. The early onset of copy-number alterations has also been reported in patients with Li-Fraumeni syndrome carrying *TP53* germline mutations [10]. Monoallelic *TP53* aberrations also induced inflammatory pathways characteristic of preleukemic states. Similar pathway upregulation has been described in heterozygous *TP53* mutant HSPCs from patients with myeloproliferative syndromes and was shown to confer a fitness advantage of *Trp53* mutant clones in experimental in vivo models [11]. *TP53* mutations have been reported to confer an immunosuppressive phenotype. We observed upregulation of the immune checkpoint ligand PD-L1 in edited HSPCs harboring monoallelic *TP53* aberrations, particularly with the R175H and R273H genotypes. PD-L1 expression has also been described in HSPCs of patients with *TP53* mutant MDS and AML where it correlates with poor survival outcomes [12]. However, disruption of a single *TP53* allele is insufficient to drive the outgrowth of UCB-derived human HSPCs. Following inactivation of the second allele, *TP53* aberrant HSPCs acquired a clonogenic growth and self-renewal advantage. The homeobox transcription factor HOXA10 was identified among upregulated genes that potentially contribute to this transition. In transgenic mice, HOXA10 has been reported to promote both proliferation and lineage commitment of hematopoietic stem cells in a concentration-dependent manner [13]. Enhanced self-renewal of biallelic mutations, considered a prerequisite for leukemic transformation, has also been described recently in a novel *Trp53* mouse model [4].

These findings provide mechanistic insights into early *TP53*-driven transformation of human HSPCs and highlight potential clinical implications. In particular, *TP53* mutant CHIP clones expand during cytotoxic therapy, ultimately giving rise to therapy-related myeloid neoplasms that are aggressive disorders associated with a dismal outcome [14]. In a recent report, patients with solid cancers and *TP53* mutant clonal hematopoiesis undergoing chemotherapy were additionally treated with the CDK4/6 inhibitors trilaciclib or palbociclib. Compared with placebo, these agents substantially mitigated the expansion of the mutant clone without causing severe side effects [15]. Given that even monoallelic *TP53* aberrations induce profound functional changes, our data support the application of such strategies targeting mutant *TP53* as early as possible—at the monoallelic CHIP stage.

## Supplementary information

Supplementary data

## Data Availability

Single-cell template strand sequencing data are deposited at the European Genome-phenome Archive (EGA) under accession number EGAS50000001965. RNA-seq data are publicly available through the European Nucleotide Archive (ENA) under accession number PRJEB101389 (BioStudy: S-BSST2246).

## References

[1] Usui Y, Endo M, Iwasaki Y, Iijima H, Nakagawa H, Matsuda K, et al. Clinical significance of TP53-mutant clonal hematopoiesis across diseases. Blood Cancer Discov. 2025;6:298–306. 10.1158/2643-3230.BCD-24-0355 PMC12209765 40525786

[2] Weeks LD, Niroula A, Neuberg D, Wong W, Lindsley RC, Luskin M, et al. Prediction of risk for myeloid malignancy in clonal hematopoiesis. NEJM Evid. 2023;2. 10.1056/evidoa2200310. PMC10361696 37483562

[3] Shah MV, Hung K, Baranwal A, Kutyna MM, Al-Kali A, Toop C, et al. Evidence-based risk stratification of myeloid neoplasms harboring TP53 mutations. Blood Adv. 2025;9:3370–80. 10.1182/bloodadvances.2024015238 PMC12277823 40085954

[4] Fullin J, Topcu E, Zielinska KA, Schimmer RR, Klemm N, Koch C, et al. The pathogenesis of therapy-related myeloid neoplasms from TP53-mutant clonal hematopoiesis. Leukemia. 2026;40:279–92. 10.1038/s41375-025-02839-5 PMC12875862 41407825

[5] Fosselteder J, Pabst G, Sconocchia T, Schlacher A, Auinger L, Kashofer K, et al. Human gene-engineered calreticulin mutant stem cells recapitulate MPN hallmarks and identify targetable vulnerabilities. Leukemia. 2023;37:843–53. 10.1038/s41375-023-01848-6 PMC10079532 36813992

[6] Loizou E, Banito A, Livshits G, Ho YJ, Koche RP, Sánchez-Rivera FJ, et al. A gain-of-function p53-mutant oncogene promotes cell fate plasticity and myeloid leukemia through the pluripotency factor FOXH1. Cancer Discov. 2019;9:962–79. 10.1158/2159-8290.CD-18-1391 PMC6606372 31068365

[7] Boettcher S, Miller PG, Sharma R, McConkey M, Leventhal M, Krivtsov AV, et al. A dominant-negative effect drives selection of TP53 missense mutations in myeloid malignancies. Science. 2019;365:599–604. 10.1126/science.aax3649 PMC7327437 31395785

[8] Wang Z, Burigotto M, Ghetti S, Vaillant F, Tan T, Capaldo BD, et al. Loss-of-function but not gain-of-function properties of mutant TP53 are critical for the proliferation, survival, and metastasis of a broad range of cancer cells. Cancer Discov. 2024;14:362–79. 10.1158/2159-8290.CD-23-0402 PMC10850947 37877779

[9] Cosenza MR, Gaiatto A, Erarslan Uysal B, Andrades A, Sautter NL, Simunovic M, et al. Origins of chromosome instability unveiled by coupled imaging and genomics. Nature. 2025;648:383–93. 10.1038/s41586-025-09632-5 PMC12695650 41162705

[10] Light N, Layeghifard M, Attery A, Subasri V, Zatzman M, Anderson ND, et al. Germline TP53 mutations undergo copy number gain years prior to tumor diagnosis. Nat Commun. 2023;14:77. 10.1038/s41467-022-35727-y PMC9816166 36604421

[11] Rodriguez-Meira A, Norfo R, Wen S, Chedeville AL, Rahman H, O’Sullivan J, et al. Single-cell multi-omics identifies chronic inflammation as a driver of TP53-mutant leukemic evolution. Nat Genet. 2023;55:1531–41. 10.1038/s41588-023-01480-1 PMC10484789 37666991

[12] Sallman, McLemore DA, Aldrich AL AF, Komrokji RS, McGraw KL, Dhawan A, et al. TP53 mutations in myelodysplastic syndromes and secondary AML confer an immunosuppressive phenotype. Blood. 2020;136:2812–23. 10.1182/blood.2020006158 PMC7731792 32730593

[13] Magnusson M, Brun AC, Miyake N, Larsson J, Ehinger M, Bjornsson JM, et al. HOXA10 is a critical regulator for hematopoietic stem cells and erythroid/megakaryocyte development. Blood. 2007;109:3687–96. 10.1182/blood-2006-10-054676 17234739

[14] Hiwase D, Hahn C, Tran ENH, Chhetri R, Baranwal A, Al-Kali A, et al. TP53 mutation in therapy-related myeloid neoplasm defines a distinct molecular subtype. Blood. 2023;141:1087–91. 10.1182/blood.2022018236 36574363

[15] Chan ICC, Zhang P, Pan X, Castro C, Fox N, Lewis AM, et al. CDK4/6 inhibition mitigates chemotherapy-induced expansion of TP53-mutant clonal hematopoiesis. Nat Genet. 2026;58:582–92. 10.1038/s41588-026-02526-w PMC12987733 41814002

