# Supplementary material for: Mutant TP53 allelic states in human hematopoietic stem and progenitor cells
Source: Leukemia. 2026 Aug 18;40(11):2602–6. doi: 10.1038/s41375-026-03106-x (PMC13713593; doi:10.1038/s41375-026-03106-x)
Supplement: Supplementary file 1 [file 41375_2026_3106_MOESM1_ESM.pdf]

Supplementary information

Mutant *TP53* allelic states in human hematopoietic stem and progenitor cells

Karin Lind, Sayantanee Dutta et al.

i) Supplementary methods

ii) Supplementary tables and figures

i) Supplementary methods

Human hematopoietic stem and progenitor cell (HSPC) isolation and culture

De-identified umbilical cord blood (UCB) was collected during full-term deliveries in collaboration with the Department of Gynecology and Obstetrics, Medical University of Graz, Austria (MUG), following written informed consent of the mothers as approved by the local ethics committee (vote number 33-534 ex 20-21). Mononuclear cells were enriched by density gradient centrifugation using Lymphoprep (StemCell Technologies, Vancouver, Canada) at 800xg for 20 minutes at room temperature (RT) with brake off. $CD34^+$ HSPCs were subsequently isolated using the CD34 MicroBead Kit (Miltenyi Biotec, Bergisch Gladbach, Germany) according to the manufacturer's instructions. Purity check after isolation was performed with CD34 FITC and CD45 V500-C (both from BD Biosciences, San Jose, CA, USA) using flow cytometry (Cytoflex LX, Beckman Coulter, Brea, CA, USA) and preparations with a purity of at least 90% were either cryopreserved in RPMI 1640 (Sigma-Aldrich/Merck/Merck, Darmstadt, Germany) supplemented with 10% FBS and 10% DMSO or used fresh (Supplementary Figure S1). For experiments, cells were cultured in serum-free stem cell retention medium (StemSpan SFEM II, StemCell Technologies) supplemented with human SCF, TPO, FLT3L, IL-6, IL-3 (all 100 ng/ml; Peprotech, Rocky Hill, NJ, USA), small molecule UM171 (35 nM, Selleckchem, Munich, Germany) and SR1 (750 nM; StemCell Technologies) and Antibiotic-Antimycotic (1x, Gibco, Carlsbad, CA, USA). Cells were maintained at a density of $0.25\text{-}1x10^6$ cells/ml for optimal growth. HSPCs from single UCB donors were used for genomic analyses while pooled HSPCs were used for all other experiments.

Editing of the TP53 gene

Our objective was to generate specific monoallelic and biallelic aberrations of the *TP53* gene: 1) The hot-spot mutation R175H, known for its gain-of-function properties, 2) the hotspot mutation R273H, which results in a dominant-negative phenotype, as well as 3) a gene knock-out (KO) (Supplementary Table S1) [1-3]. To achieve this, we employed a knock-in strategy using CRISPR/Cas9 in combination with recombinant adeno-associated virus serotype 6 (rAAV6), as previously described [4-6]. As a *TP53* wild-type (WT) control, we targeted the *AAVS1* safe harbor locus by knocking-in the fluorescent reporter cassettes [5, 7].

AAV vector plasmids were cloned into the pAAV-MCS plasmid (Agilent Technologies, Foster City, CA, USA), which contains inverted terminal repeats (ITRs) from AAV serotype 2. To introduce the hotspot mutations, we generated *TP53* targeting vectors containing full-length codon optimized *TP53* cDNAs (Integrated DNA Technologies) carrying either the mutation of interest (R175H, c.524G>A; R273H, c.818G>A) or the WT sequence. To create KO alleles, an additional codon optimized cDNA introducing a premature stop codon in exon 2 (c.22T>A) was generated. The vectors also included fluorescent reporter genes (GFP, BFP, or mCherry) under the control of a separate SFFV promotor enabling enrichment of successfully modified genes using flow cytometry. The right and left homology arms, flanking the cDNA, were 244 bp and 250 bp, respectively (Supplementary Figure 2A). rAAV6 vectors were produced as described previously [4]. Briefly, HEK293T cells (DSMZ, Braunschweig, Germany, ACC 635) were expanded and seeded at a density of 1×10^7 cells per dish into ten 15 cm dishes. The following day, the expansion medium was replaced with transfection medium consisting of DMEM supplemented with 10% FBS, 1 mM sodium pyruvate, 25 mM HEPES, 1×L-glutamine, and 1 mM sodium butyrate (all from Sigma-Aldrich/Merck/Merck). For the transfection of ten dishes, 10 ml of Opti-MEM (Gibco) were mixed with 60 µg of ITR-containing AAV donor plasmids, 220 µg of the helper plasmid pDGM6 (Addgene, Watertown, MA, USA), and 1120 µl of polyethylenimine (PEI; Polysciences, Hirschberg, Germany). After 72 hours of incubation in a humidified incubator, cells were harvested, and rAAV6 vectors were purified using the AAVpro Purification Kit (TaKaRa, Shiga, Japan) according to the manufacturer's instructions. Viral vectors were quantified using digital droplet PCR (QX200, Bio-Rad, Hercules, CA, USA), aliquoted, and stored at -80 °C until further use. HEK293T cells were authenticated by short tandem repeat (STR) profiling using the PowerPlex 16HS System (Promega, Madison, USA). Routine mycoplasma testing was performed with the Venor GeM Mycoplasma Detection Kit (BioProducts, Stockerau, Austria) by the Core Facility 'Alternative Biomodels and Preclinical Imaging' (Center for Medical Research, MUG).

Synthetic single-guide (sg) RNAs, chemically modified at their three terminal nucleotides with 2'-O-methyl-phosphorothioate bonds to gain more stability and efficiency, were purchased from Synthego (Redwood City, CA, USA). The sgRNA *TP53* MUT1 (5'-TCGACGCTAGGATCTGACTG-3', PAM: CGG) targeted the *TP53* locus 12 bases downstream of the start codon, enabling the insertion of the codon-optimized cDNAs for either *TP53* WT or the mutations of interest into the correct endogenous regulatory context. The sgRNA *AAVS1*

(5´-GGGGCCACTAGGGACAGGAT-3´, PAM: TGG) was used to target the safe harbor locus. Cas9 protein with a nuclear localization signal (Alt-R Cas9 Nuclease V3, NLS) was purchased from Integrated DNA Technologies (IDT, Coralville, IA, USA). Prior to electroporation, ribonucleoprotein (RNP) complexes were prepared by incubating 15 µg Cas9 protein with 8 µg sgRNA (1:2.5 molar ratio) at 25 °C for 10 minutes. Electroporation was performed using a 4D Nucleofector (Lonza, Basel, Switzerland) with the program DZ-100 and the P3 Primary Cell 4D-Nucleofection Kit according to the manufacturer's instructions. Cells (2×10^5 - 1×10^6) were pelleted (300 × g, 5 minutes, RT), resuspended in 20 µl (2×10^5 cells) or 100 µl (1×10^6 cells) nucleofection solution containing the RNP complex, and immediately supplemented with pre-warmed cytokine-rich StemSpan SFEM II (Stemcell Technologies) to achieve 0.25 - 0.5×10^6 cells/ml. rAAV6 donor vectors were added at a multiplicity of infection of 5,000 - 7,000 within 20 minutes post-electroporation, and medium was replaced after 6 - 8 hours to remove residual virus. Seventy-two hours later, cells were sorted by high GFP/BFP or mCherry/BFP expression (FACSAria IIIu, BD Biosciences) to enrich edited cells with integrated donor vectors. Sorted cells were used immediately or expanded in StemSpan SFEMII for downstream analyses.

Confirmation of site-specific knock-in

Reporter-positive, genome-edited cells or individual colonies picked from CFU assays, were transferred into 25 µl QuickExtract lysis buffer (Illumina, Epicentre, San Diego, California, USA) or DNA was extracted using either the QiAamp DNA Micro or Mini Kit (Qiagen, Hilden, Germany), depending on the total cell number. Site-specific integration of the *TP53* cDNA and the fluorescent reporter cassette was detected by "In-PCR" using a forward primer binding inside the left homology arm and a reverse primer specific to the codon-optimized *TP53* cDNA (1,298 bp). Lack of integration was revealed by "Out-PCR" using a reverse primer binding outside of the right homology arm in the WT sequence (552 bp) (Supplementary Figure 2B). PCR amplification was performed using the HotStarTaq Master Mix Kit (Qiagen). Amplicons specific for the insertion were separated via gel electrophoresis, purified (New England Biolabs, Frankfurt am Main, Germany), and subjected to Sanger sequencing (ABI 3730XL instrument, TubeSeq, Eurofins Genomics, Ebersberg, Germany) to confirm seamless donor integration using CodonCodeAligner software (vs 4.0.4, Codoncode Corporation, Centerville, USA). To confirm correct integration at the RNA level, mRNA was extracted using mRNeasy

Micro Kit (Qiagen) reverse transcribed from 1 µg of total RNA with gene specific primers using High Capacity cDNA Reverse Transcription Kit (Thermo Fisher Scientific, Waltham, MA, USA). The resulting cDNA was amplified by PCR using *TP53* specific primers and analyzed by Sanger sequencing. All primer sequences are available upon request.

Immunocytochemical staining of p53 expression

Reporter-positive, genome-edited HSPCs were treated with 10 µM Nutlin-3 (Sigma-Aldrich/Merck) for 24 hours. DMSO (WAK Chemie, Steinbach, Germany) was used as control. Aliquots of 50 µl cell suspension, containing $1x10^4$ - $4x10^4$ cells, were mixed with 100 µl DPBS and centrifuged with 600xg for 3 minutes at RT in a Cytospin Centrifuge (Thermo Fisher Scientific) onto SuperFrost® Plus slides (Menzel Epredia, Thermo Fisher Scientific). Air-dried cytospin slides were fixed using 4% neutral-buffered formaldehyde (Cell Signaling, Danvers, Massachusetts, USA) for 10 minutes. Pre-treatment and immunocytochemical staining using 3,3′-diaminobenzidine (DAB) as chromogen were performed on an automated platform (Dako Omnis, Agilent, Santa Clara, CA, USA). Heat-induced epitope retrieval was done using EnVision FLEX Target Retrieval Solution High pH (Dako), at 95°C for 24 minutes. Staining with the p53 antibody (clone DO-7, mouse anti-human, binding at amino acids 1 - 45; Dako), targeting both WT and aberrant p53, was performed for 20 minutes. For visualization, slides were incubated with EnVision FLEX detection kit according to the manufacturer´s instructions and counterstained with hematoxylin (both Dako). After the staining, slides have been mounted with permanent mounting method. Whole-slide imaging was performed using the Aperio slide scanner ScanScope AT (Leica Biosystems, Wetzlar, Germany) at the Zentrum für Center for Medical Research, Medical University of Graz and data analysis was performed using QuPath software version 0.5.1 (open-source, University of Edinburgh, Edinburgh, UK). DAB positivity was quantified based on mean optical density, and cells were classified as DAB-positive or -negative using a predefined threshold that was kept constant across all samples.

Western blot analysis of p53 expression

Reporter-positive, genome-edited OCI-AML5 cells (DSMZ, ACC 247) were treated with DMSO (WAK Chemie), 10 µM Nutlin-3 (Sigma-Aldrich/Merck), or 100 nM daunorubicin (DNR; Selleckchem) for 24 hours to induce p53. Cells ($5 \times 10\^{5}$–$1 \times 10\^{6}$) were resuspended in 50–100 µl RIPA buffer (Sigma-Aldrich/Merck) supplemented with 1× phosphatase and protease

inhibitors (Thermo Fisher Scientific) and lysed by three freeze-thaw cycles in liquid nitrogen. Following centrifugation at 17,000 × g for 5 minutes at 4°C, whole-cell extracts (WCE) were quantified using the Lowry protein assay (Bio-Rad). Samples were mixed with 2× or 4× Laemmli buffer (Bio-Rad) and denatured at 90°C for 5 minutes. Equal amounts of protein (20 µg) were separated on 4–15% Mini-PROTEAN® TGX™ Precast gels using 1× TGS running buffer at 100 V for 1 hour in a Mini-PROTEAN chamber (Bio-Rad). Proteins were transferred onto PVDF membranes using 1× TG transfer buffer containing methanol at 400 mA for 1 hour at 4°C. Membranes were blocked in 5% non-fat dry milk in TBS-T for 1 hour at room temperature and incubated overnight at 4°C with primary antibodies against p53 (1:5,000; Sigma-Aldrich/Merck, #P6874) and GAPDH (1:5,000; Santa Cruz Biotechnology, sc-32233). After washing three times for 5 minutes with TBS-T, membranes were incubated with HRP-conjugated secondary antibodies (horse anti-mouse, 1:3,000, #7076; goat anti-rabbit, 1:10,000, #7074S; Cell Signaling Technology). Detection was performed using WesternBright ECL substrate (Advansta) and a ChemiDoc Touch imaging system with Quantity One software (Bio-Rad). Authentication of OCI-AML5 cells was carried out using the PowerPlex 16HS System (Promega, Madison, USA), and absence of mycoplasma contamination was verified with the Venor GeM Mycoplasma Detection Kit (BioProducts, Stockerau, Austria). Both analyses were conducted by the Core Facility 'Alternative Biomodels and Preclinical Imaging' (Center for Medical Research, MUG).

5-ethynyl-2'deoxyuridine (EdU) proliferation assay

Reporter-positive, genome-edited HSPCs were cultured in StemSpan SFEM II medium supplemented with additives as described above. Proliferation analysis was performed on day 3 using the Click-iT® Plus EdU Alexa Fluor 488 Flow Cytometry Assay Kit (Thermo Fisher Scientific). Between $0.2x10^5$ and $2x10^5$ cells were seeded into 48-well plates. After 24 hours, 5 µM EdU was added for 3 hours. Cells were then harvested, washed, fixed and permeabilized according to the manufacturer's instructions. Detection is based on a so called 'click reaction' - a chemical reaction between the incorporated EdU and Alexa Fluor picolyl azide. DNA content was measured using FxCycle Far Red Staining Solution (Thermo Fisher Scientific), and the percentage of cells in different cell cycle phases was determined by flow cytometry (Cytoflex LX, Beckman Coulter).

Colony-forming unit (CFU) assays

Reporter positive, genome-edited HSPCs were sorted into StemSpan SFEM II medium and mixed with methylcellulose medium (Methocult, StemCell Technologies). Cells were seeded at a density of 300 cells per dish in 35 mm dishes. After 14 days of incubation at 37°C, colonies were counted and classified as burst-forming unit-erythroid (BFU-E), colony-forming unit-macrophage (CFU-M) or colony-forming unit granulocyte/macrophage (CFU-GM), according to the manual of StemCell Technologies. For serial replating of CFU assays, between 3,000 and 100,000 cells from pooled colonies were washed with DPBS (Gibco), resuspended in IMDM (Sigma-Aldrich/Merck), and subsequently seeded into methylcellulose medium.

Single-cell template strand sequencing

TP53 aberrations are preferentially associated with structural copy number variations [8]. We investigated these alterations in our human HSPC model following doxorubicin treatment of edited HSPCs using single-cell template strand sequencing. This technology enables resolution of individual homologs within a single cell by selectively analyzing the template DNA strands [9, 10].

Initially we determined the optimal doxorubicin concentration for this approach and assessed phosphorylated H2AX (γH2AX) and Annexin V as markers of DNA damage and apoptosis, respectively. Therefore, unedited HSPCs were expanded in StemSpan SFEMII medium supplemented with additives as described above (StemCell Technologies) and seeded into 48-well plates at a density of $2x10^5$ cells per 500 µl. Doxorubicin (Selleckchem) was added at concentrations ranging from 10 nM - 150 nM, 24 hours post-seeding. After 48 hours of treatment, cells were harvested, centrifuged at 300xg for 7 minutes, and washed twice with ice cold DPBS. Between $2x10^5$ and $1x10^6$ cells were then fixed in 100 µl of 4% formaldehyde (Cell Signaling) for 15 minutes at RT. For permeabilization, cells were washed with excess DPBS and resuspended in 100 µl Cell Permeabilization Buffer (Cell Signaling) for 10 minutes at RT. Following centrifugation, cells were incubated with the Phospho-Histone H2A.X antibody (Alexa Fluor 488; Cell Signaling) or the isotype control (Alexa Fluor IgG XP; Cell Signaling) at a 1:100 dilution. Between $1x10^5$ and $5x10^5$ were resuspended in 100 µl of diluted antibody solution (DPBS with 0.5% BSA) and incubated for 1 hour at +4°C, protected from light. Cells were then washed twice, resuspended in 200 µl DPBS, and analyzed for γH2AX expression by flow cytometry using a Cytoflex LX (Beckman Boulter). To assess apoptosis, $2x10^5$ - $1x10^6$ cells

were harvested 48 hours following doxorubicin treatment, centrifuged at 300xg for 7 minutes and washed twice with ice-cold DPBS. Cells were resuspended in 100 µl Annexin V binding buffer, stained with 0.5 µl Annexin V FITC (Biolegend, San Diego, CA, USA), and incubated for 10 - 15 minutes at RT. Subsequently, 100 – 400 µl Annexin V binding buffer was added, and the percentage of Annexin V-positive cells was determined by flow cytometry using Cytoflex LX. These data revealed that doxorubicin treatment at 10 nM is sufficient to induce DNA damage. However, the formation of chromosomal abnormalities (CAs) needs sufficient time for cell divisions to occur. We therefore treated cells with 10 nM doxorubicin for 7 days followed by a 3-day recovery period. This condition yielded sufficient γH2AX induction while maintaining minimal apoptosis levels and cell loss in HSPCs (Supplementary Figure S6).

Single-cell template strand sequencing was performed as previously described [9, 10] and depicted in Supplementary Figure 7. Reporter-positive, genome-edited HSPCs were expanded for 21 to 28 days and seeded at a density of $2x10^5$ per well in 500 µl SFMII complete medium (StemCell Technologies). On the following day, either 10 nM doxorubicin (Selleckchem) or DMSO (WAK Chemie) was added for 7 days. After medium replacement, HSPCs were re-seeded at the same density and allowed to recover for 3 days. Cells were then labelled with 40 µM bromodeoxyuridine (BrdU; Sigma-Aldrich/Merck) for 24, 36 and 48 hours. At each time point, the reaction was stopped by adding DPBS, and the suspensions were transferred to 50 ml Falcon tubes. Cells were counted, centrifuged at 300xg for 4 minutes at RT and frozen with 1 ml of freezing medium at -80°C for downstream analyses. Frozen cells were thawed in a +37°C water bath, transferred to Falcon tubes containing 2 ml of ice-cold DPBS, counted and centrifuged again at 300xg for 4 minutes at RT. Nuclei Staining Buffer A was prepared by adding 1 ml of 1 M Tris-HCl (Thermo Fisher Scientific), 308 µl of 5 M NaCl, 10 µl of 1 M $CaCl_2$, 5 µl of 1 M $MgCl_2$ (all Sigma-Aldrich/Merck), 267 µl of 7.5% BSA solution (Thermo Fisher Scientific), 100 µl of 10% NP-40-Alternative (Sigma-Aldrich/Merck) and 10 µl of 10 mg/ml Hoechst 33258 (Thermo Fisher Scientific) to 8.3 ml of endotoxin-free ultrapure water (Sigma-Aldrich/Merck), yielding a total volume of 10 ml. Cells were resuspended at $1x10^6$/ml, avoiding vortexing, and extracted nuclei were kept on ice and protected from light. Unstained nuclei - without Hoechst dye - served as negative controls. TheraPEAK™ ProFreeze™ CDM (Lonza) was prepared in 5 ml PBS containing 1x ProFreeze Buffer and 7.5% DMSO (v/v). A volume of 5 µl was dispensed into each well of a 96-well PCR plate (hard-shell Armadillo; Thermo Fisher Scientific). Plates were sealed with SILVERseal aluminium foil (Greiner Bio-One,

Kremsmünster, Austria) and kept on ice. To avoid nuclear clumping and dye bleaching, one sample at a time was thawed and immediately sorted into the prepared plates using a FACSAria IIIu (BD Biosciences). A BrdU-negative sample, corresponding to the undivided nuclei population, was used to set the negative gate. BrdU-positive (divided) nuclei - displaying approximately half the Hoechst fluorescence intensity - were sorted into individual wells into nuclei lysis buffer. After sorting, plates were centrifuged in a pre-chilled centrifuge at 500xg for 5 minutes and stored at -80°C. Thawed nuclei were subjected to a specific sequencing library protocol. Chromatin was digested by an MNase protocol. Chromatin was digested by an MNase to generate mononucleosomes, blunt-ended fragments were 5'-phosphorylated and 3' adenylated using commercially available kits (New England Biolabs, Frankfurt, Germany), followed by ligation of specific paired-end adaptors (Illumina). BrdU-labeled DNA strands were selectively removed by Hoechst-UV treatment: samples were incubated with Hoechst dye for 15 minutes and then irradiated under UV light for an additional 15 minutes. This step nicked BrdU-substituted strands, enabling selective PCR amplification of the original, BrdU-negative template strands. Sequencing libraries were pooled and indexed with unique hexamer barcoded primers (Illumina). DNA clean-up steps were performed using paramagnetic beads and a magnetic stand (Beckman Coulter). Paired-end sequencing (75 bp reads) was carried out on a NextSeq 5000 platform (Illumina), using custom primers to read the hexamer barcode. Reads were aligned to GRCh38 reference genome using BWA-MEM (v0.7.17) and processed with MosaiCatcher tool to detect copy number alterations across various ploidy backgrounds. Read depth, strand orientation (Watson/Crick ratios), and haplotype information per nucleus were analyzed with scTRIP. This computational pipeline enabled the classification of structural variants into chromosome losses and gains, either interstitial, terminal or multistep, inverted duplications, complex chromosomal aberrations and chromothripsis, respectively, as previously described [11].

Transcriptome analysis

Reporter-positive, genome-edited HSPCs were expanded for three weeks, followed by a second cell sorting step. Cells were resuspended in QIAzol according to pellet size (450 µl for $<1 \times 10^6$ cells and 700 µl for $\geq 1 \times 10^6$ cells). Total RNA was extracted using the miRNeasy Kit, including on-column DNase digestion with the RNase-Free DNase Set (Qiagen). Cells were homogenized by vortexing for 1 minutes 30 sec, and chloroform was added at 0.2 volumes.

RNA concentration was measured using a NanoDrop 2000 spectrophotometer (Thermo Fisher Scientific), and RNA integrity was assessed with the RNA 6000 Pico LabChip on a 2100 Bioanalyzer (Agilent), yielding RNA integrity numbers (RIN) ranging from 6.4 to 9.2. The low RNA output resulting from low cell numbers led to the use of low-input polyA-amplification protocol for transcriptomic next-generation sequencing and generates full-length cDNA prior to library construction. RNA-seq libraries were prepared from 5 ng total RNA using the NEBNext Single Cell/Low Input RNA Library Prep Kit for Illumina® (New England Biolabs), with SPRIselect beads (Beckman Coulter) used for purification at all steps. cDNA was amplified for 12 cycles, and 20 ng amplified cDNA were used for library construction with adapters diluted 1:15. Final library amplification was performed for 10 cycles. All samples were processed together and prepared as a single sequencing library to minimize batch effects. Library concentrations were quantified using a CLARIOstar plate reader (BMG Labtech, Ortenberg, Germany) with the QuantiFluor ONE dsDNA System (Promega, WI, Madison, USA), and fragment size distribution was analyzed using the DNA High Sensitivity LabChip on the 2100 Bioanalyzer (Agilent), confirming a mean fragment size of approximately 280 bp. An additional 0.9× bead-based purification of the pooled libraries was performed to remove adapter and primer dimers. Sequencing was carried out on a NovaSeq 6000 system (Illumina) using 100 bp paired-end reads, generating 28–38 million reads per sample (Vienna BioCenter Core Facilities, Vienna, Austria). After demultiplexing, FASTQ files were generated for downstream analysis. Quality control of raw files was assessed by using FastQC (version 0.11.9). (https://www.bioinformatics.babraham.ac.uk/projects/fastqc/) followed by preprocessing which included length and quality filtering and quality based trimming. Raw reads were then mapped to the Genome Reference Consortium human genome GRCh38 assembly using STAR (Spliced Transcripts Alignment to a Reference, version 2.7.9a) [12]. Aligned reads were processed and sorted using SAMtools (version 1.4) [13]. Gene-level read counts based on the UCSC RefSeq (refGene) annotation were obtained utilizing featureCounts (version 2.0.3) [14] from the Subread package. Normalization and differential expression analysis were carried out using the DESeq2 package in R [15]. Volcano plots were generated by GraphPad Prism version 9.2.0. (GraphPad Software, San Diego, CA, USA). Differentially expressed genes were considered significant with an adjusted p-value <0.05 and log^2 fold change $> \pm 1$. Venn diagrams were created using DeepVenn (https://www.deepvenn.com/). In addition, gene set enrichment analysis (GSEA, version 4.3.3; Broad Institute) was performed on the normalized

expression data of the DESeq2 package [16] . Gene sets were obtained from the Molecular Signatures Database (MSigDB 2020), including Hallmark gene sets, as well as curated erythroid and myeloid gene signatures derived from a human haematopoietic atlas [17]. Values with a false discovery rate (FDR) < 0.25 were considered significant. Normalized enrichment scores (NES) and corresponding enrichment plots were generated and reported.

PD-L1 expression analysis

HSPCs were expanded for 4 days prior to CRISPR/Cas9-mediated gene editing. Three days post editing, double fluorescent reporter-positive cells were sorted and further expanded for up to 3 weeks in StemSpan SFEM II medium supplemented according to standard conditions. PD-L1 expression was analyzed by flow cytometry on days 21, 22, and 23 post sorting. For analysis, 100 µL cell suspension was collected from each well of a 48-well plate and replaced with fresh culture medium. Cells were then transferred to Eppendorf tubes and centrifuged at 300×g for 7 minutes at RT. The supernatant was discarded and HSPCs were stained using a master mix prepared in staining buffer (DPBS supplemented with 1% BSA and 0.2 mM EDTA). Per reaction, 50 µL staining buffer, 2.5 µL Fc receptor blocking reagent (BioLegend, Cat# 422302), and either 0.5 µL anti PD-L1 APC antibody (400 µg/mL, clone 29E.2A3, BioLegend, Cat# 329708) or 2.0 µL mouse IgG2b APC isotype control antibody (100 µg/mL, BioLegend, Cat# 401210) were added, resulting in a total staining volume of 50 µL per sample. Cells were incubated for 20 minutes at 4°C protected from light, followed by washing with at least 800 µL staining buffer. Cells were transferred to FACS tubes and centrifuged at 300×g for 7 minutes at RT, and the supernatant was removed. Flow cytometric acquisition was performed on a CytoFLEX LX (Beckman Coulter), acquiring equal numbers of cells per sample (5,000 events). Data was analyzed using FlowJo (BD Biosciences). Unstained edited HSPCs were included as fluorescence minus one (FMO) controls to define negative gating thresholds. The percentage of PD-L1 positive cells was determined based on isotype control gating.

Statistical analysis

Data were expressed as mean ± SEM. Pairwise comparisons between groups were performed using a two-sided, paired Student's t-test. For nominal data, Chi^2 test or Fisher's exact test was used. Beta regression analysis was performed in R (version 4.3.2) and R studio. For multiple-comparison analysis either one-way or two-way ANOVA with post hoc Tukey's multiple

comparison was performed using Graphpad Prism software version 9.2.0. (GraphPad Software). Data with statistical significance are indicated with asterisks: ns, $P \geq 0.05$; *$P < 0.05$; **$P < 0.01$; ***$P < 0.001$; ****$P < 0.0001$.

References

1. Loizou E, Banito A, Livshits G, Ho YJ, Koche RP, Sánchez-Rivera FJ*, et al.* A Gain-of-Function p53-Mutant Oncogene Promotes Cell Fate Plasticity and Myeloid Leukemia through the Pluripotency Factor FOXH1. *Cancer Discov* 2019;**9:**962-979.

2. Boettcher S, Miller PG, Sharma R, McConkey M, Leventhal M, Krivtsov AV*, et al.* A dominant-negative effect drives selection of TP53 missense mutations in myeloid malignancies. *Science* 2019;**365:**599-604.

3. Wang Z, Burigotto M, Ghetti S, Vaillant F, Tan T, Capaldo BD*, et al.* Loss-of-Function but Not Gain-of-Function Properties of Mutant TP53 Are Critical for the Proliferation, Survival, and Metastasis of a Broad Range of Cancer Cells. *Cancer Discov* 2024;**14:**362-379.

4. Bak RO, Dever DP, Porteus MH. CRISPR/Cas9 genome editing in human hematopoietic stem cells. *Nat Protoc* 2018;**13:**358-376.

5. Bak RO, Dever DP, Reinisch A, Cruz Hernandez D, Majeti R, Porteus MH. Multiplexed genetic engineering of human hematopoietic stem and progenitor cells using CRISPR/Cas9 and AAV6. *Elife* 2017;**6:**

6. Fosselteder J, Pabst G, Sconocchia T, Schlacher A, Auinger L, Kashofer K*, et al.* Human gene-engineered calreticulin mutant stem cells recapitulate MPN hallmarks and identify targetable vulnerabilities. *Leukemia* 2023;**37:**843-853.

7. Oceguera-Yanez F, Kim SI, Matsumoto T, Tan GW, Xiang L, Hatani T*, et al.* Engineering the AAVS1 locus for consistent and scalable transgene expression in human iPSCs and their differentiated derivatives. *Methods* 2016;**101:**43-55.

8. Rucker FG, Dolnik A, Blatte TJ, Teleanu V, Ernst A, Thol F*, et al.* Chromothripsis is linked to TP53 alteration, cell cycle impairment, and dismal outcome in acute myeloid leukemia with complex karyotype. *Haematologica* 2018;**103:**e17-e20.

9. Falconer E, Hills M, Naumann U, Poon SS, Chavez EA, Sanders AD*, et al.* DNA template strand sequencing of single-cells maps genomic rearrangements at high resolution. *Nat Methods* 2012;**9:**1107-1112.

10. Sanders AD, Falconer E, Hills M, Spierings DCJ, Lansdorp PM. Single-cell template strand sequencing by Strand-seq enables the characterization of individual homologs. *Nat Protoc* 2017;**12:**1151-1176.

11. Cosenza MR, Rodriguez-Martin B, Korbel JO. Structural Variation in Cancer: Role, Prevalence, and Mechanisms. *Annu Rev Genomics Hum Genet* 2022;**23:**123-152.

12. Dobin A, Davis CA, Schlesinger F, Drenkow J, Zaleski C, Jha S*, et al.* STAR: ultrafast universal RNA-seq aligner. *Bioinformatics* 2013;**29:**15-21.

13. Li H, Handsaker B, Wysoker A, Fennell T, Ruan J, Homer N*, et al.* The Sequence Alignment/Map format and SAMtools. *Bioinformatics* 2009;**25:**2078-2079.

14. Liao Y, Smyth GK, Shi W. featureCounts: an efficient general purpose program for assigning sequence reads to genomic features. *Bioinformatics* 2014;**30:**923-930.

15. Love MI, Huber W, Anders S. Moderated estimation of fold change and dispersion for RNA-seq data with DESeq2. *Genome Biol* 2014;**15:**550.

16. Subramanian A, Tamayo P, Mootha VK, Mukherjee S, Ebert BL, Gillette MA*, et al.* Gene set enrichment analysis: a knowledge-based approach for interpreting genome-wide expression profiles. *Proc Natl Acad Sci U S A* 2005;**102:**15545-15550.

17. Granja JM, Klemm S, McGinnis LM, Kathiria AS, Mezger A, Corces MR*, et al.* Single-cell multiomic analysis identifies regulatory programs in mixed-phenotype acute leukemia. *Nat Biotechnol* 2019;**37:**1458-1465.

ii) Supplementary tables and figures

Table S1. *TP53* constructs used for gene editing, their induced mutant allelic state and functional consequences. Abbreviations: mCh, monomeric Cherry fluorescent protein; BFP, blue fluorescent protein; GFP, green fluorescent protein.

***TP53* cDNA donor combinations**	***TP53* allelic state**	**p53 functionality**
Mutant R175H $^{mCh\ or\ GFP}$ /WT^{BFP}	monoallelic	gain-of-function
Mutant R175H $^{mCh\ or\ GFP}$ /KO^{BFP}	biallelic	gain-of-function
Mutant R273H $^{mCh\ or\ GFP}$ /WT^{BFP}	monoallelic	dominant-negative
Mutant R273H $^{mCh\ or\ GFP}$ /KO^{BFP}	biallelic	loss-of-function
KO $^{mCh\ or\ GFP}$ /WT^{BFP}	monoallelic	haploinsufficiency
KO $^{mCh\ or\ GFP}$ /KO^{BFP}	biallelic	loss-of-function

Table S2. Uniquely upregulated genes in monoallelic *TP53* aberrant HSPCs (n = 70). Abbreviation: P_{adj}, adjusted P-value

Gene symbol	Log2FoldChange	Padj
LOC284080	6.446383567	0.045944225
RASD2	6.339698619	0.03028012
HTR1B	6.091010336	0.041436984
ELFN1	6.07192805	0.014246829
FAM183BP	5.61529184	0.008366582
SH2D1A	4.945533318	0.024114549
PPBP	4.320628276	0.044044308
NAP1L2	4.282247743	0.045068023
ORM2	3.606355643	0.01880349
CCL18	2.960922565	0.012089835
DCSTAMP	2.649923074	0.001539231
FGFR2	2.466452616	0.024164555
ANOS1	2.326514942	0.021271174
LOC653786	2.260097677	0.009094883
CA12	2.237745962	0.030665354
SNORA11	2.215686805	0.035624272
GAMT	2.208964799	0.009874075
GRIN2C	2.204357035	0.02919211
CHI3L2	2.176871035	0.034193209
WNK4	2.118468985	0.023582496
AKR1C3	2.068904987	0.048649311
CETN4P	2.028742804	0.036874398
CXCL8	2.023059878	0.026220092
MIR3945HG	1.7969138	0.04029632
CDR2L	1.756479089	0.01192065
LOC105376805	1.754392628	0.03973573
SLIT3	1.749451764	0.009679544
LOC90246	1.741243158	0.013059708
CYP27A1	1.734761757	0.000377931
FGFBP3	1.705183862	0.019185718
RN7SK	1.683419915	0.018084202
PLPP3	1.678451582	0.036485502
JAG2	1.657894731	0.045472526
ZMIZ1-AS1	1.614065377	0.00038569
RNU12	1.590360696	0.038397449
C3	1.534992146	0.008238266
ATP2C1	1.525244112	0.00370186
CD274	1.512260901	0.005115345
TRIM7	1.465297206	0.02149818
FXYD6	1.459075294	0.010330836
TMEM236	1.425106297	0.021821434
GAPLINC	1.399254773	0.003863881
CDC37L1-AS1	1.391733304	0.043964685
AGRP	1.364815907	0.022026542
CR1	1.235005725	0.032277153
PRKX-AS1	1.231798713	0.046572911
OSM	1.229705559	0.04413656
DLGAP1-AS1	1.229607627	0.001952277
MAPRE3	1.227686334	0.048463263
SDC4	1.224903581	0.001184975
CDC14B	1.198684458	0.02746687
MRC1	1.189171022	0.025529625
LINC01272	1.184939596	0.020625541
CD69	1.176140095	0.017667065
SIAE	1.159391445	0.010687364
JAKMIP2	1.153638749	0.002391302
FAM27B	1.141666005	0.046132888
IFI30	1.134471161	0.000224548
RAB11FIP4	1.108469861	0.046132888
RPS27L	1.098350816	6.11064E-05
NPL	1.089609185	0.000452573
RDH10	1.072259747	0.041436984
CD300LF	1.05742015	0.02746687
PET100	1.052197923	0.005669792
C15orf52	1.051019509	0.010330836
LOC100507006	1.036400199	0.011745945
TP53TG1	1.036297364	0.021663258
LAMTOR2	1.018883475	0.034891784
CXCR4	1.013155315	0.028908831
NDUFB1	1.007438084	0.022824653

Table S3. Uniquely upregulated genes in biallelic *TP53* aberrant HSPCs (n = 30). Abbreviation: P_{adj}, adjusted P-value

**Gene symbol**	**Log² fold change**	**P_{adj}**
AR	4.862448824	0.032814772
LOC729224	4.293246329	0.041686047
KCNJ12	3.770710843	0.0197177
VGLL3	3.253031483	0.016055357
IL1RAPL1	3.141262677	0.017939745
ZNF702P	2.440218735	0.011528368
TBX1	2.131665374	0.01267653
IGSF9B	1.939120572	0.007214472
PRSS33	1.894015198	0.022481344
GAL3ST4	1.597073448	0.008207256
PATJ	1.55698844	0.010945281
LINC01314	1.517438164	0.042109579
VENTX	1.508333895	0.022633151
RNF138P1	1.339497093	0.018363086
ATP10D	1.302628781	0.02674022
COL9A2	1.275565851	0.015910472
PROX2	1.235677842	0.025321011
PRSS3	1.228609666	0.025932895
ZNF284	1.102568722	0.033956748
SESN1	1.09401506	3.99695E-06
LOC100130264	1.092587903	0.021215916
LOC646471	1.038553136	0.012690768
NAALADL1	1.023648894	0.012909427
CCDC122	1.017270045	0.018958584
TEC	1.016526577	0.003915846
GSTT1	1.015368395	0.015608122
MAGEH1	1.009330503	0.002621665
LOC389906	1.008056882	0.018379715
CDK15	1.005103545	0.036895209
HOXA10	1.002742991	0.024703209

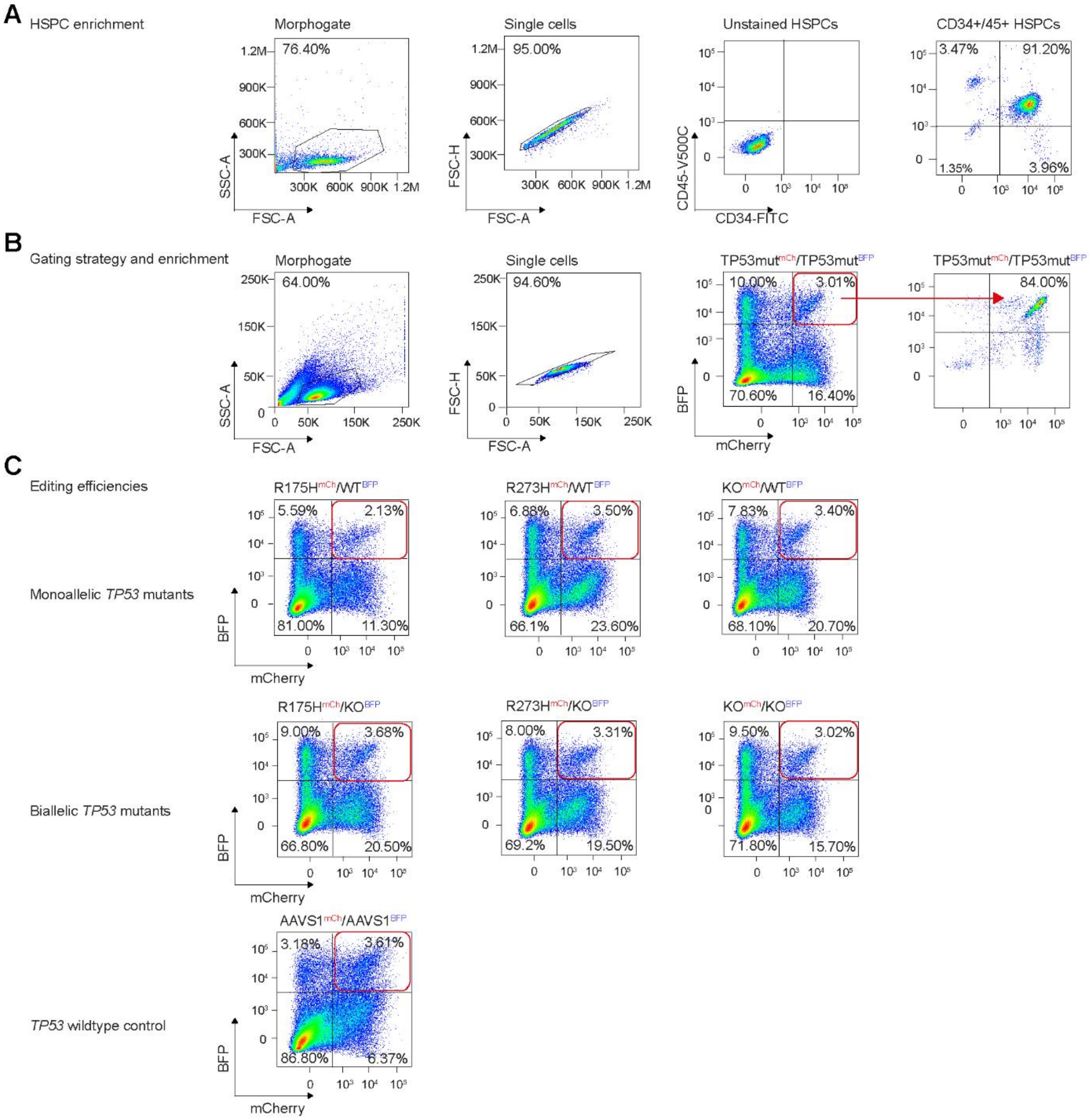

Figure S1. Flow cytometric gating strategy, assessment of enrichment and editing efficiencies of CD34$^+$ human HSPCs. (A) Cell debris was excluded using forward scatter area (FSC-A) versus side scatter area (SSC-A) plots (morphology gate), doublets were excluded using FSC-A versus forward scatter height (FSC-H) plots (single cells). (B) CD34$^+$ and CD45$^{+\ dim}$ staining confirmed enrichment of magnetically isolated HSPCs; unstained controls were used to define negative gates. Dual-fluorescent reporter-positive (mCherry$^+$/BFP$^+$, red box) population was sorted to enrich for edited HSPCs, achieving >80% purity for *TP53* mutants. (C) Representative flow cytometry plots showing editing efficiencies (red boxes) in HSPCs carrying either monoallelic (R175H/WT, R273H/WT, KO/WT) or biallelic (R175H/KO, R273H/KO, KO/KO) *TP53* aberrations, as well as edited *AAVS1* safe harbor control. Overall editing efficiencies ranged from 0.5 -10.0% (mean ± s.d., 2.23 ± 1.76).

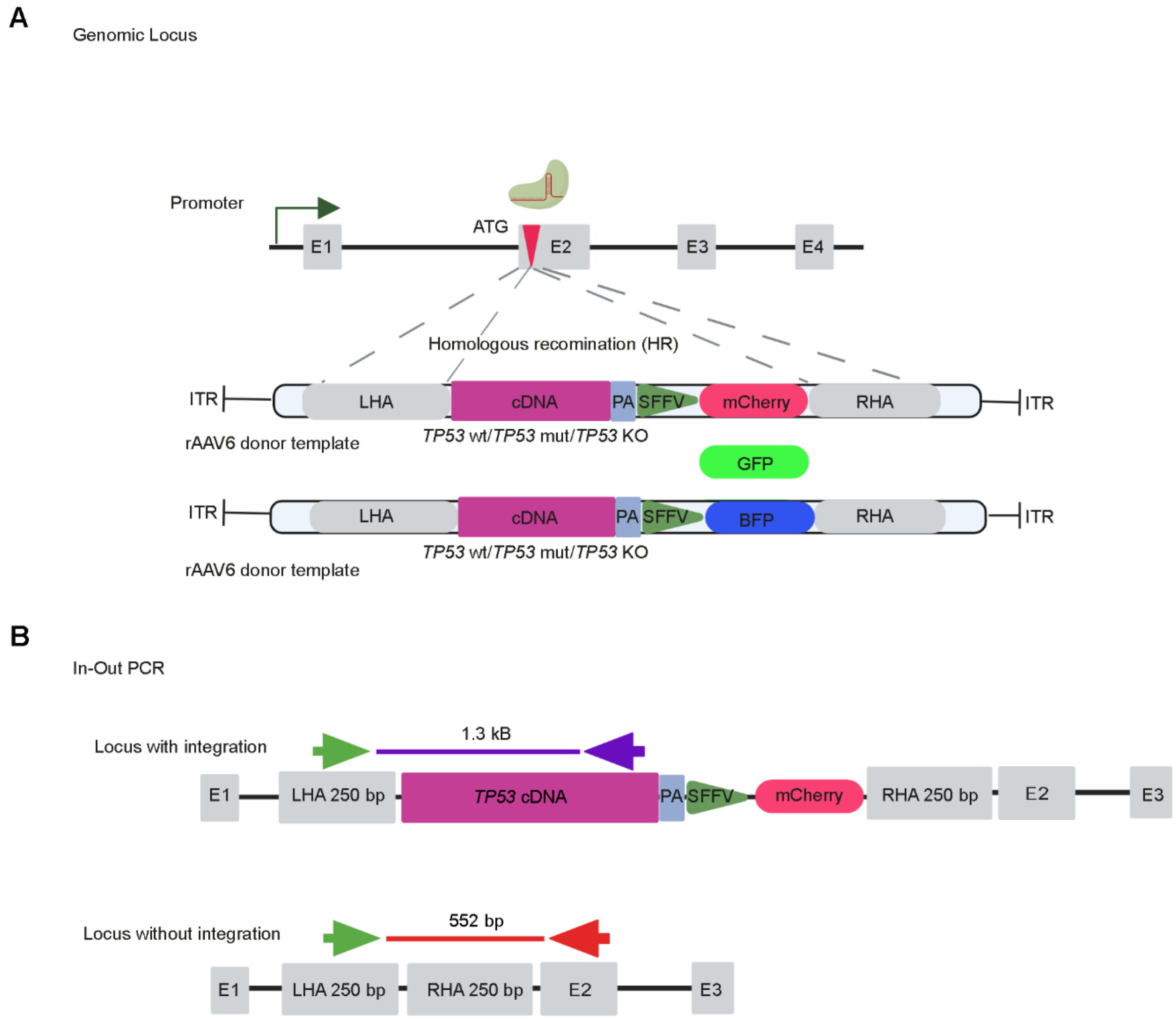

Figure S2. *TP53* targeting strategy and donor validation. (A) Schematic illustration of CRISPR/Cas9-mediated targeting of *TP53* exon 2. The sgRNA directs Cas9 cleavage 12 bp downstream of the ATG start codon. The donor template contains a 250 bp left homology arm (LHA), codon-optimized *TP53* cDNA, SV40 polyA signal, SFFV promoter, a fluorescent reporter (mCherry, GFP, or BFP), and a 244 bp right homology arm (RHA). (B) Site-specific integration was confirmed by In-Out PCR using a forward primer in the LHA and a reverse primer in the donor sequence (1.3 kb product, purple arrow). A control PCR with a reverse primer outside the RHA detected the wild-type allele (552 bp, red arrow). ITR, inverted terminal repeats; E1-E4, *TP53* exons 1–4.

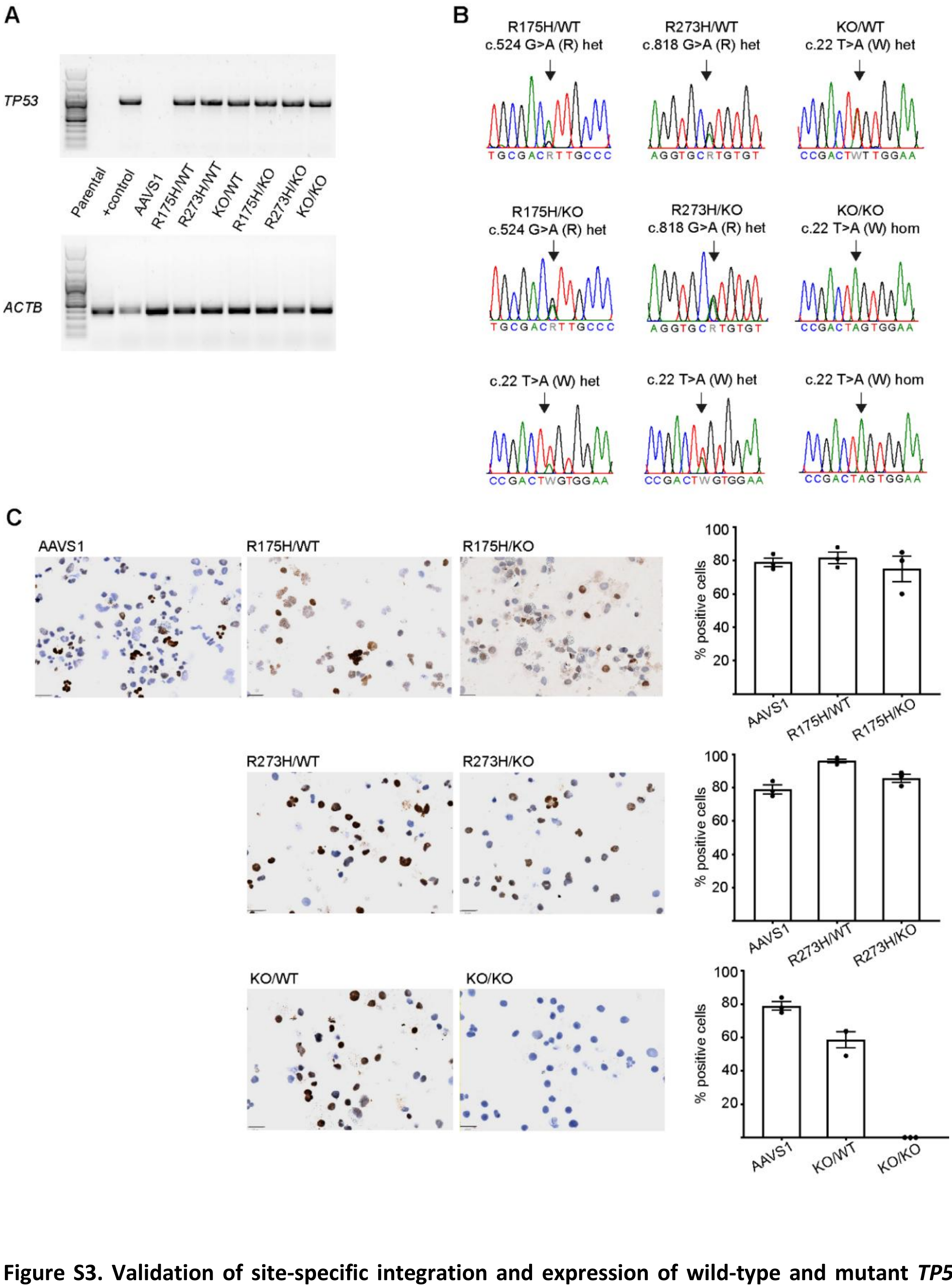

Figure S3. Validation of site-specific integration and expression of wild-type and mutant *TP53* in engineered human HSPCs. (A) PCR amplification of a 775 bp fragment confirmed integration of mutant *TP53* cDNA; no product was detected in unedited parental or *AAVS1* control cells (upper agarose gel). *ACTB* (382 bp) served as internal PCR control (lower agarose gel). (B) Sanger chromatograms show the specific *TP53* mutations in cDNA (black arrow). (C) Immunocytochemical p53 expression and quantification in *AAVS1* control HSPCs as well as HSPCs with monoallelic (R175H/WT, R273H/WT, KO/WT) and biallelic (R175H/KO, R273H/KO) *TP53* aberrations showing p53 expression whereas KO/KO clones lacked detectable expression after Nutlin-3 treatment (10 µM, 24 hours). Percentage of DO-7 positive nuclei (brown staining) was quantified using Aperio ScanScope AT slide scanner and QuPath software.

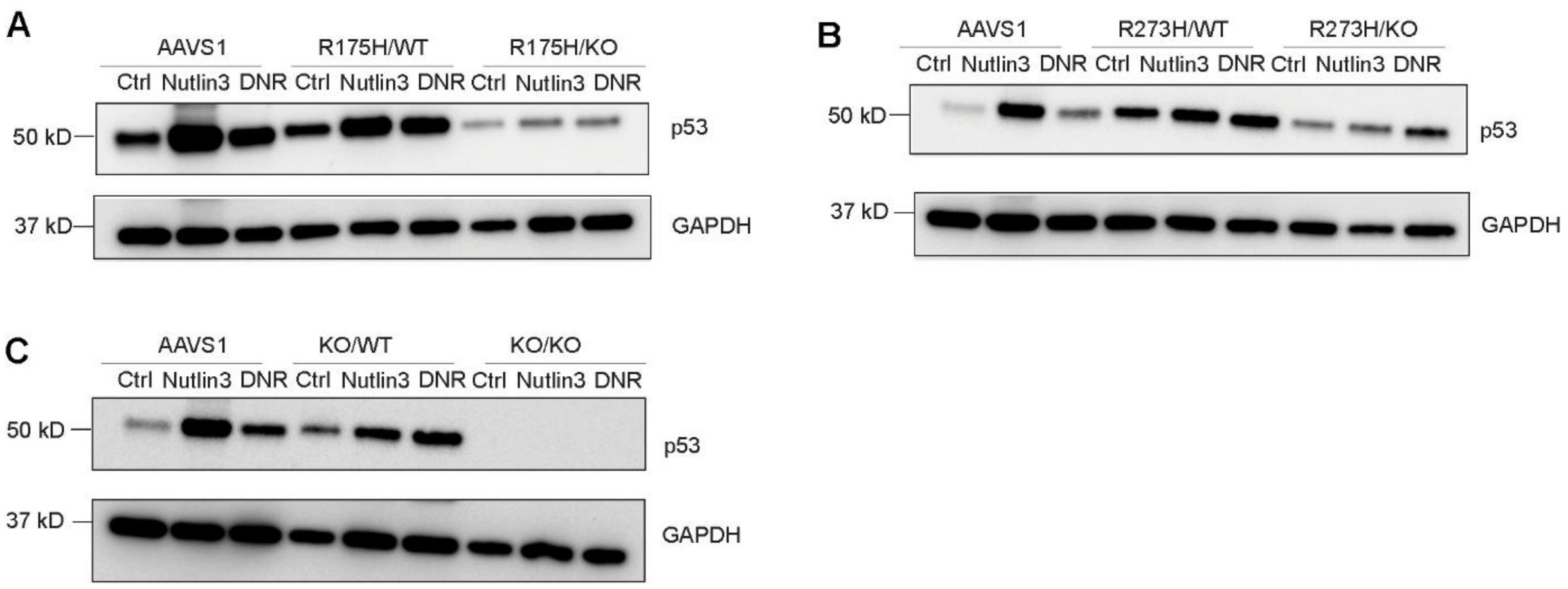

Figure S4. p53 pathway activation in the myeloid OCI-AML5 cell line. Western blot analysis of AAVS1 safe harbor control and OCI-AML5 cells harboring monoallelic or biallelic *TP53* aberrations following treatment with Nutlin-3 (10 nM) or daunorubicin (DNR, 100 nM) for 24 hours. DMSO was used as vehicle control. Each lane was loaded with 20 µg of whole-cell lysate. Primary antibodies used were anti-p53 (53 kDa) and anti-GAPDH (37 kDa) as loading control. Expression of p53 following treatment with Nutlin-3 or DNR in AAVS1 wild-type control cells and in (A) R175H mutants (R175H/WT, R175H/KO), (B) R273H mutants (R273H/WT, R273H/KO), and (C) KO/WT and KO/KO cells.

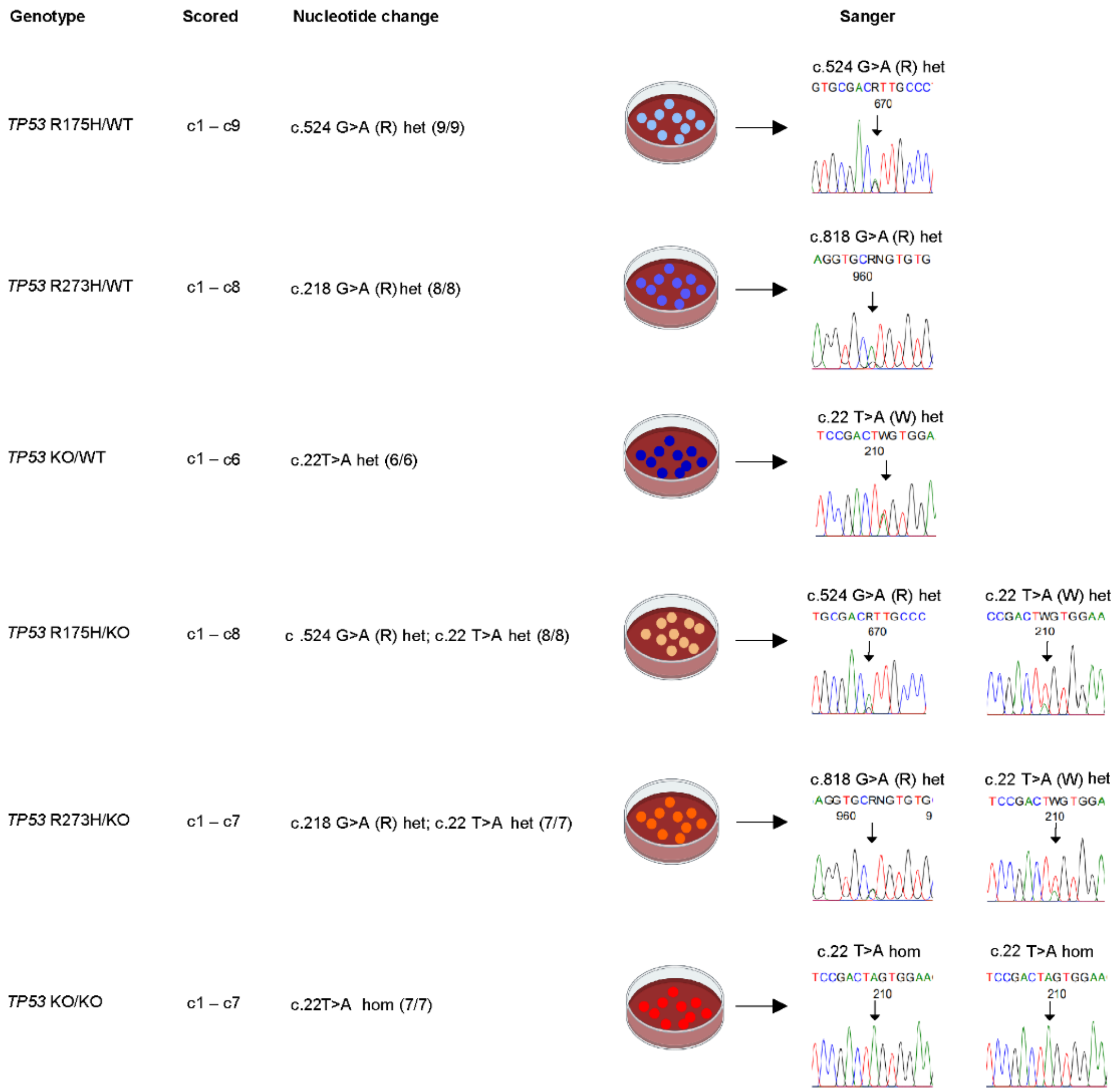

Figure S5. Genotype confirmation of single-cell derived colonies. Reporter-positive, CRISPR/Cas9 edited HSPCs were seeded in methylcellulose, individual colonies were picked and analyzed after 14 days. Sanger sequencing confirmed monoallelic (R175H/WT, R273H/WT, KO/WT) and biallelic (R175H/KO, R273H/KO, KO/KO) *TP53* mutant genotypes. Representative chromatograms are shown; arrows indicate edited nucleotides.

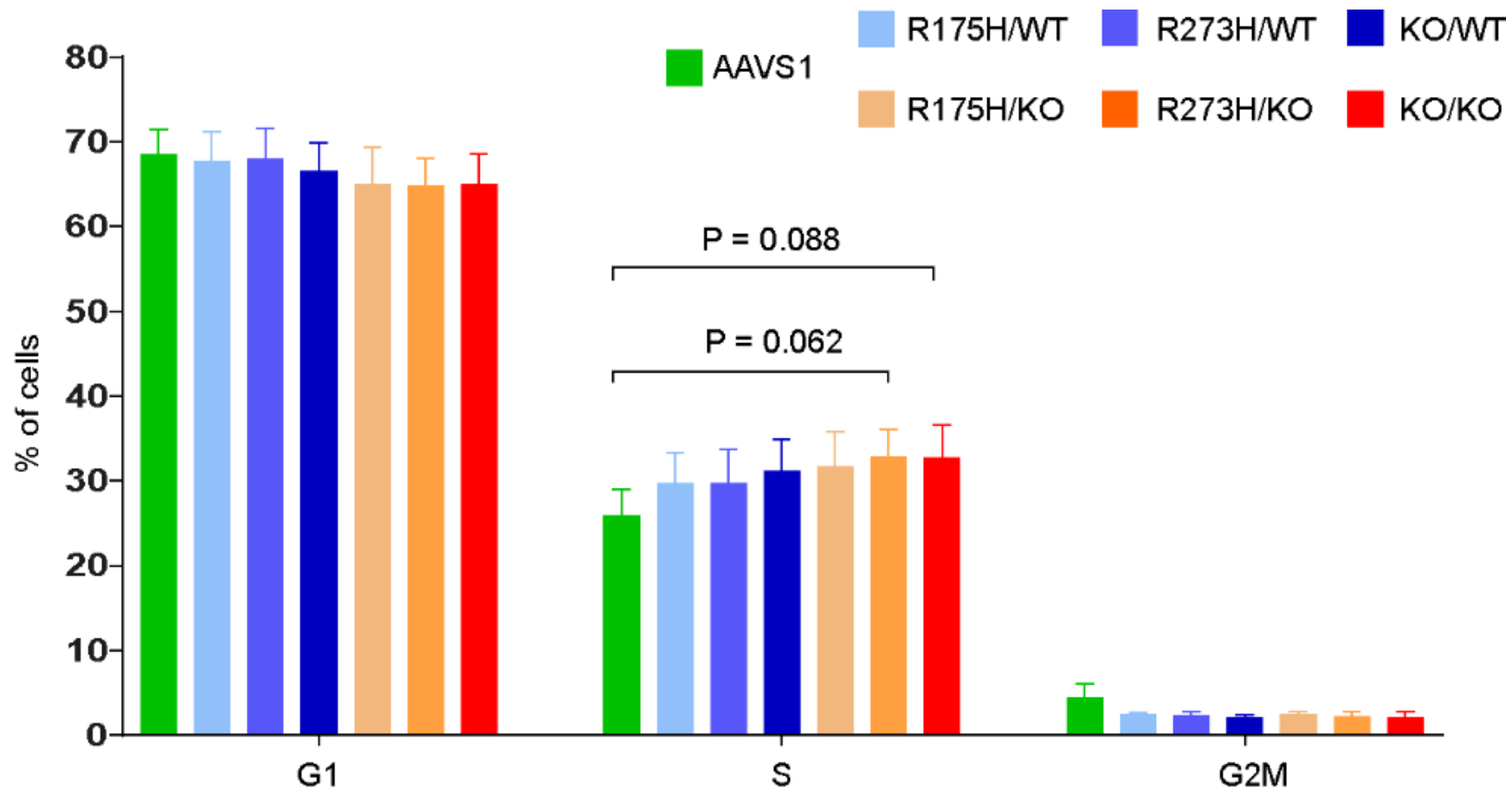

Figure S6. Increased S-phase fraction of human HSPCs harboring biallelic *TP53* mutants. Bar plots show cell-cycle distribution of HSPCs with the specific *TP53* aberrations and *AAVS1* controls (EdU assay, day 3 post-sort). Biallelic R273H/KO and KO/KO clones displayed an increased S-phase fraction of borderline significance compared to monoallelic mutants and control cells. Data represent mean ± s.e.m. from three independent experiments using three biological replicates (donor pools); P values were calculated using beta regression analysis.

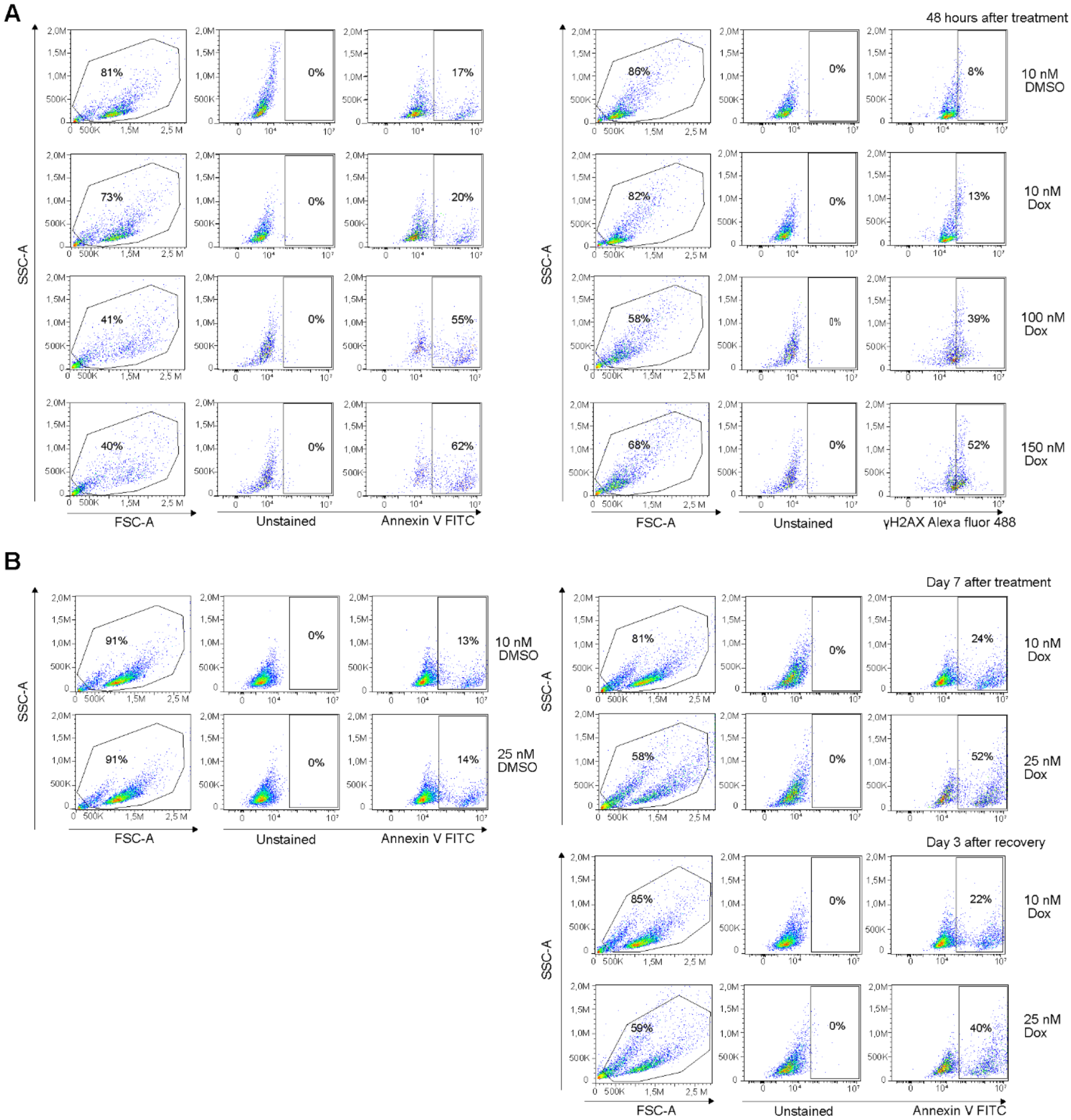

Figure S7. Determination of the optimal doxorubicin concentration for single-cell template strand sequencing. (A) Flow cytometric analysis of HSPCs treated for 48 h with DMSO or increasing concentrations of doxorubicin (Dox, 10, 100, and 150 nM). Apoptotic cells (Annexin V$^+$) increased from 17% (DMSO) to 20%, 55%, and 62%, while DNA damage (γH2AX$^+$) increased from 8% to 13%, 39%, and 52%, respectively. (B) FACS analysis of HSPCs treated for 7 days with 10 nM or 25 nM doxorubicin or DMSO, followed by a 3-day recovery period. Apoptosis rates were 10.6% and 14% in DMSO-treated samples, 24% and 52% after doxorubicin exposure, and 22% and 40% after recovery. Unstained HSPCs were used to define negative gates.

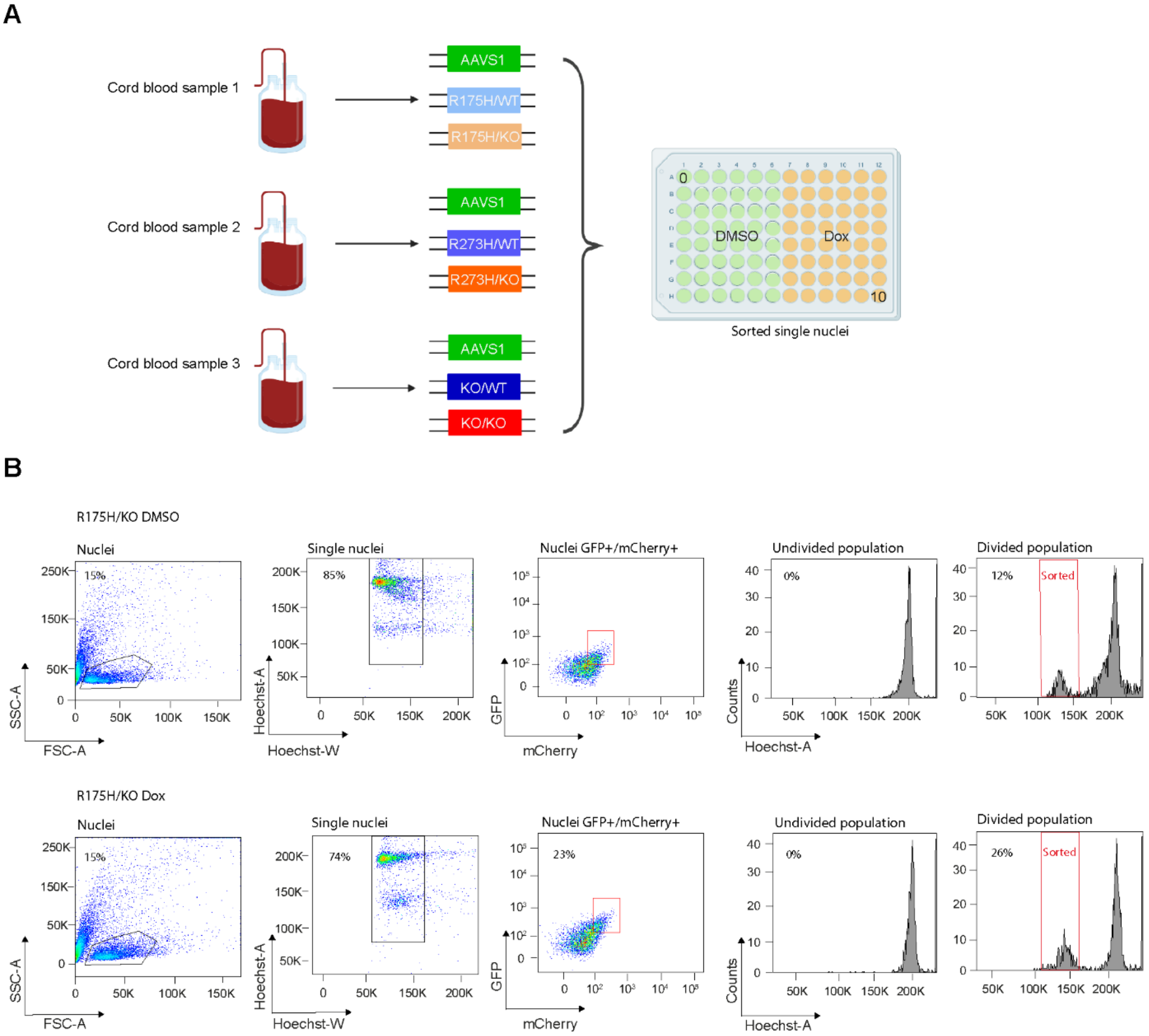

Figure S8. Experimental setup and gating strategy for single-cell template strand sequencing. (A) CRISPR-edited HSPCs were generated from a single cord blood sample to introduce *TP53* mutations R175H/WT, R175H/KO, R273H/WT, R273H/KO and heterozygous or homozygous knockouts, modeling monoallelic and biallelic *TP53* alterations. A reporter knock-in at the AAVS1 locus in the same cord blood sample served as *TP53* wild-type control. For each genotype, 47 nuclei treated with DMSO or doxorubicin were individually sorted into wells of a 96-well plate. Well A1 contained a negative control (no nuclei), and well H12 a positive control (10 nuclei). Figure created with BioRender.com (B) Representative FACS plots of *TP53* mutant (R175H/KO) single nuclei sort (upper panel: DMSO condition; lower panel: doxorubicin condition). Debris was excluded based on FSC-A versus SSC-A, and doublets were removed using Hoechst-W versus Hoechst-A. BrdU-labeled, reporter-high ($GFP^+/mCherry^+$) nuclei were gated on the population with quenched Hoechst fluorescence (red gate), corresponding to divided nuclei. A BrdU-negative control was used to define the gate for undivided nuclei with full Hoechst fluorescence.

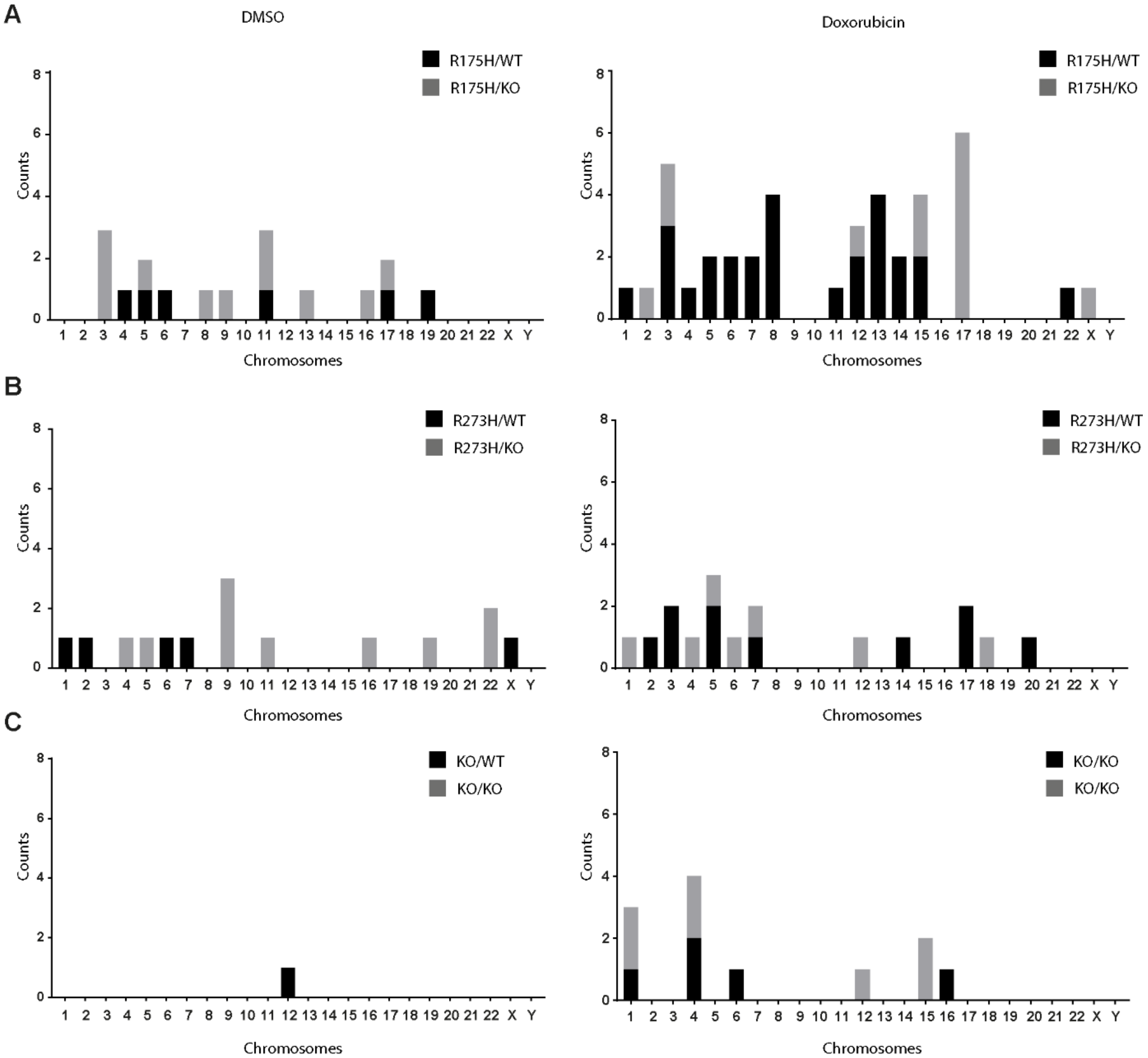

Figure S9. Chromosomal distribution of chromosomal aberrations in *TP53*-mutant human HSPCs. Bar plots show the number of chromosomal aberrations (CAs) per chromosome in monoallelic and biallelic *TP53* mutants: (A) R175H/WT and R175H/KO, (B) R273H/WT and R273H/KO, and (C) KO/WT and KO/KO. Cells were treated with DMSO (left) or doxorubicin (right).

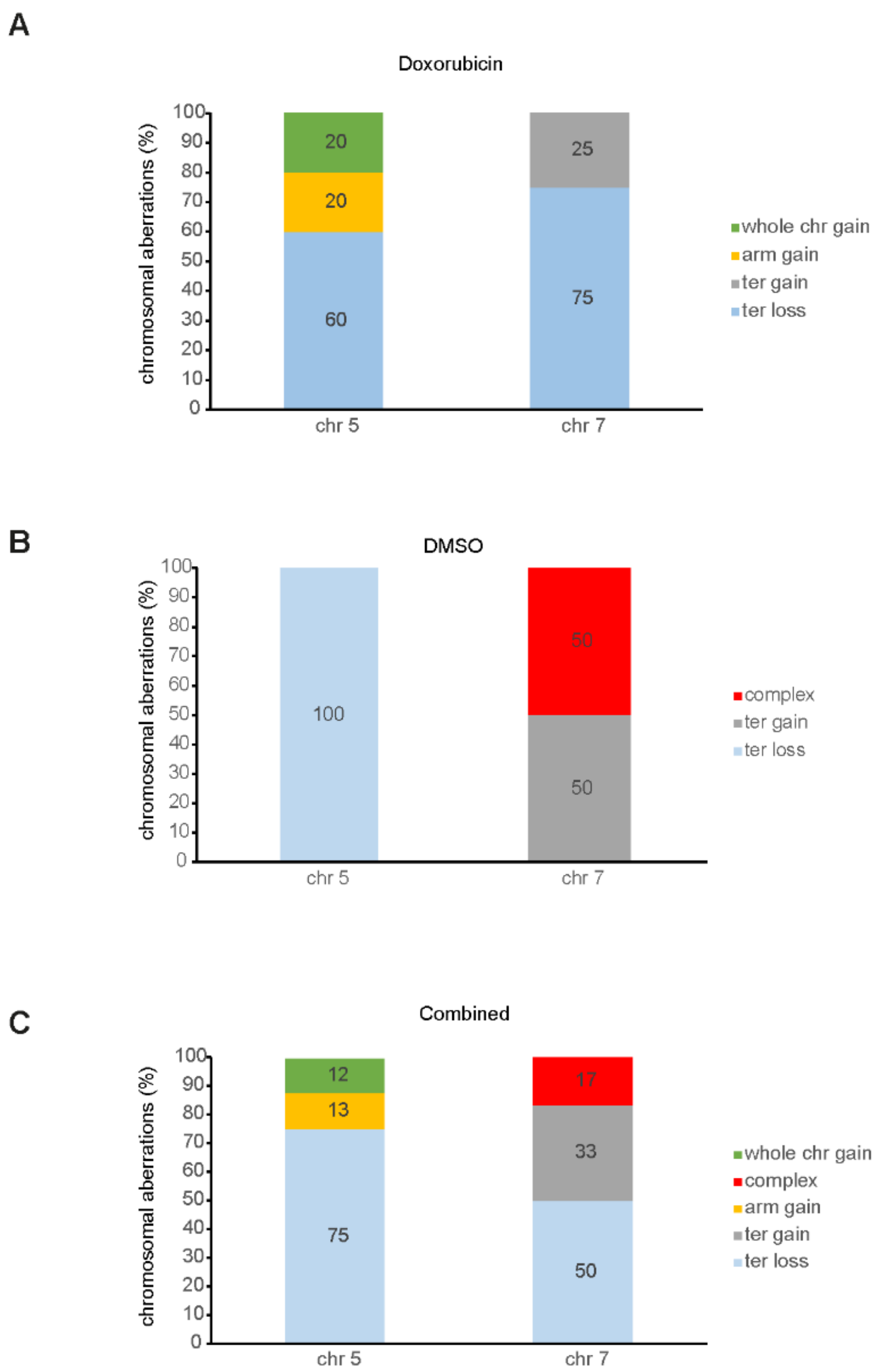

Figure S10. Spectrum of chromosomal aberrations in recurrently affected chromosomes 5 and 7 in *TP53*-aberrant human HSPCs. Stacked bar plots show the distribution of chromosomal aberrations affecting chromosomes 5 and 7. (A) Doxorubicin-treated HSPCs. (B) DMSO-treated control HSPCs. (C) Combined conditions. Values indicate the percentage of each aberration type relative to all aberrations observed per chromosome. Chromosomal aberrations are classified as whole chromosome gain, chromosome arm gain, terminal gain, terminal loss, or complex aberrations. Terminal events refer to sub-chromosomal alterations involving distal chromosomal regions. Abbreviations: chr, chromosome; ter, terminal.

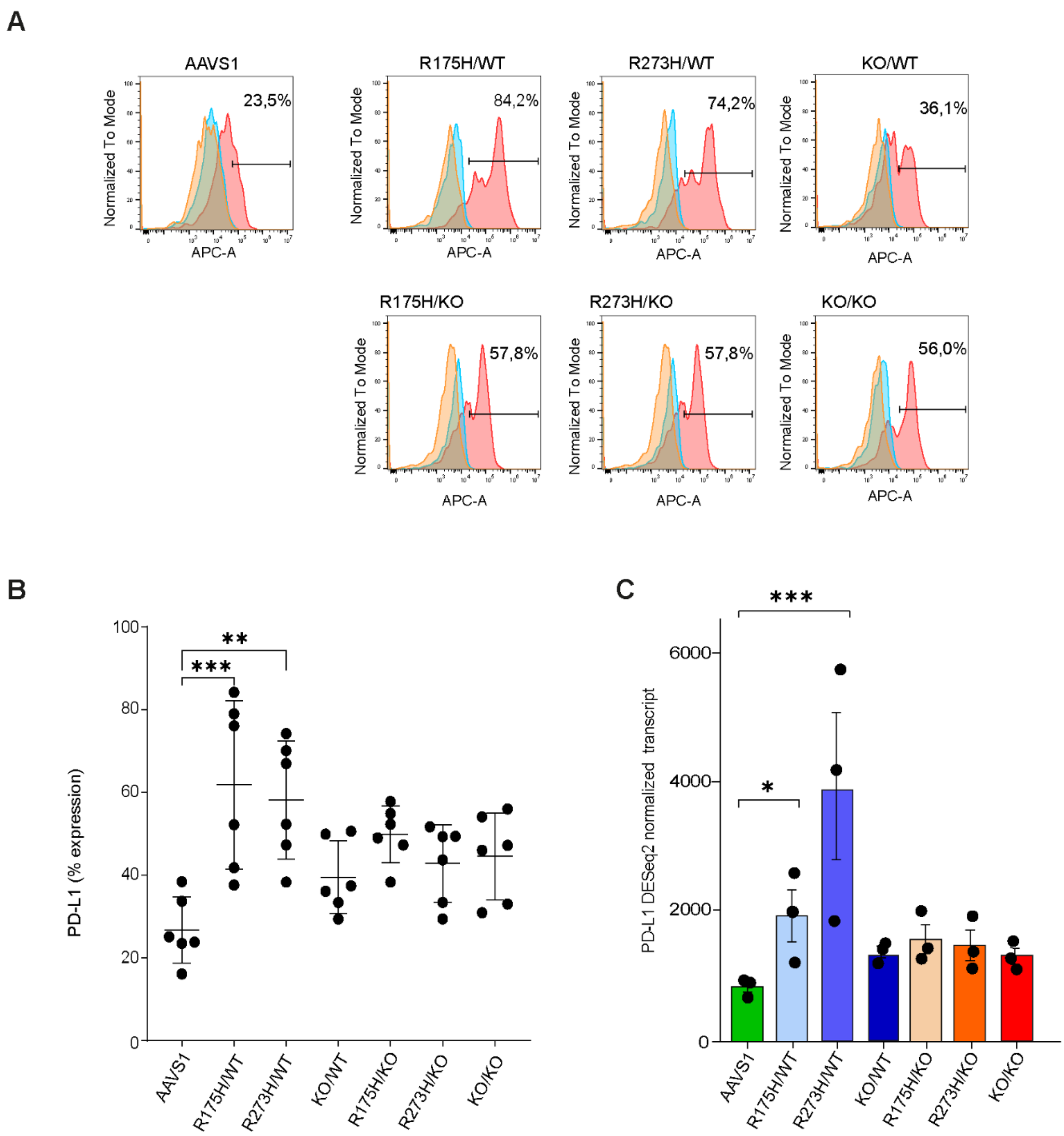

Figure S11. PD-L1 expression of human HSPCs with *TP53* aberrant genotypes. (A) Representative flow cytometry histograms showing PD-L1 surface expression (APC-A) in the indicated genotypes. Histograms are normalized to mode to enable comparison of distribution profiles. Red histograms represent PD-L1 staining, blue indicates the isotype control (mouse IgG), and orange represents unstained control. Numbers indicate the percentage of PD-L1 positive cells. (B) The percentage of *PD-L1*-positive cells was determined using isotype control–based gating. Data points represent measurements obtained at three timepoints (days 21, 22, and 23), in line with the transcriptome analysis, from two different samples (#340 and #367). Statistical comparisons were performed between all genotypes using one-way ANOVA followed by Tukey's multiple-comparisons test. Bars indicate mean ± s.e.m. (C) Bar plot showing mean DESeq2-normalized counts of PD-L1 transcript levels ± s.e.m. for each *TP53* genotype and AAVS1 control and individual data points are overlaid (n=3). Statistical analysis was performed using one-way ANOVA followed by Dunnett's multiple comparisons test against the AAVS1 control. *$p < 0.05$, **$p < 0.01$, ***$p < 0.001$

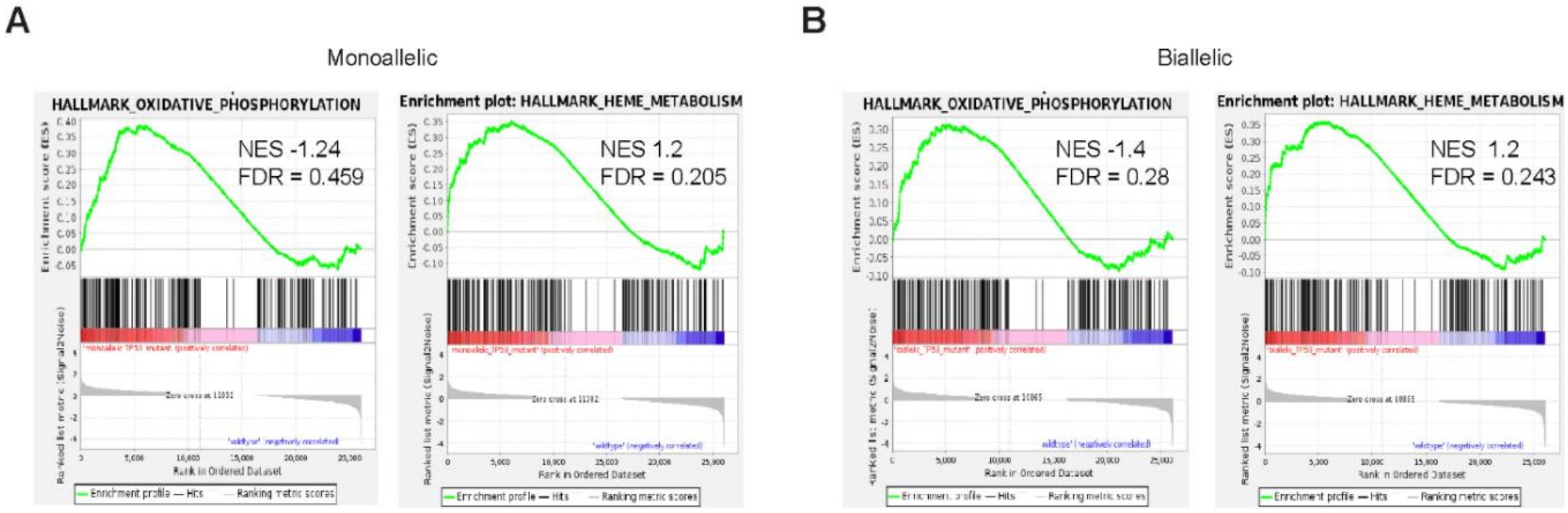

Figure S12. Gene set enrichment analysis (GSEA) of the HALLMARK_OXIDATIVE_PHOSPHORYLATION and the HALLMARK_HEME_METABOLISM gene sets in monoallelic and biallelic *TP53* aberrant human HSPCs compared to wild-type controls. Both monoallelic (A) and biallelic (B) *TP53* mutant cells show enrichment of oxidative phosphorylation and heme metabolism pathway genes.

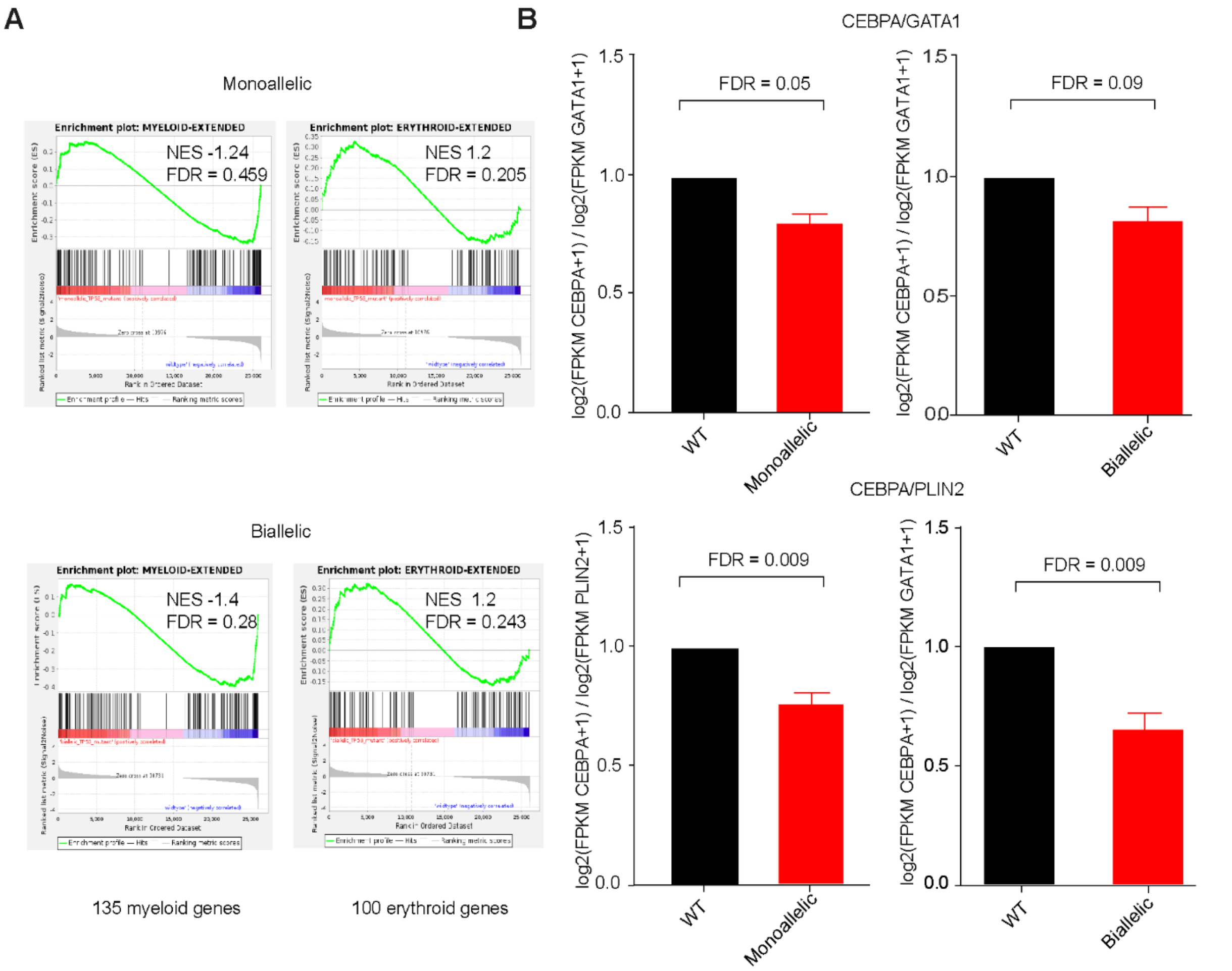

Figure S13. Myeloid and erythroid gene expression signatures in *TP53* mutant human HSPCs. (A) GSEA plots showing negative enrichment of 135 myeloid genes (NES = −1.4, FDR > 0.25) and positive enrichment of 100 erythroid genes (NES = 1.2, FDR < 0.25) in HSPCs with monoallelic (upper panel) and biallelic (lower panel) *TP53* aberrations. (B) Ratio of *CEBPA* to the erythroid marker *GATA1* and *PLIN2* transcripts in AAVS1 control (WT *TP53*) as well as in monoallelic and biallelic *TP53* mutant HSPCs. FDR values were adjusted using the Benjamini-Hochberg method following an unpaired t-test or Mann–Whitney test. Abbreviation: GSEA, gene set enrichment analysis.
